# Computational Mechanistic Insights on the NO Oxidation Reaction Catalyzed by Non-Heme Biomimetic Cr-N-Tetramethylated Cyclam Complexes

**DOI:** 10.3390/ijms20163955

**Published:** 2019-08-14

**Authors:** Tiziana Marino, Maria Grazia Fortino, Nino Russo, Marirosa Toscano, Marta Erminia Alberto

**Affiliations:** Dipartimento di Chimica e Tecnologie Chimiche, Università della Calabria, 87036 Rende, Italy

**Keywords:** biomimetic chromium catalysts, DFT, potential energy surface, NO catalytic conversion

## Abstract

The conversion reaction of NO to NO_3_^−^ ion catalyzed by the end-on [Cr(III)(n-TMC)(O_2_)(Cl)]^+^ superoxo and side-on [Cr(IV)(n-TMC)(O_2_)(Cl)]^+^ peroxo non-heme complexes (*n* = 12, 13, 14 and 15), which are biomimetic systems of nitric oxide dioxygenases (NODs), has been explored using a computational protocol in the framework of density functional theory. Results show that the potential energy profiles for the studied reactions lie above the reagent energies, regardless of the used catalyst. Both the O-O bond breaking in the biomimetics and the NO_3_^−^ ion formation require low energy barriers suggesting an efficient catalytic power of the studied systems. The rate-determining step depends on ligand size.

## 1. Introduction

Oxidation reactions play an important role in different fields from biochemistry to industrial processes to organic synthesis. 

In many metabolic processes, oxidation of organic substrates by dioxygen (O_2_) is mediated by metal containing active sites, therefore, a great deal of attention has been paid to the catalytic cycles of O_2_ activation by heme and non-heme metalloenzymes [1]. The considerable interest towards heme iron-oxy catalytic species lies in obtaining information which is also transportable to non-heme natural and synthetic complexes. Recent advances in synthetic non-heme model chemistry have allowed to gain insights about the reactivity and the structural and physicochemical properties of the metal-oxygen intermediates, generated in the active sites of different oxidases and oxygenases [2]. 

With the aim to highlight the mechanism of nucleophilic and electrophilic oxidation reactions catalyzed by various oxygenases, different bioinspired analogues have been synthesized and characterized using the wide technical arsenal of modern chemistry [3,4]. Of particular interest appear to be the studies on mononuclear metal complexes, including chromium, iron, cobalt, nickel and copper ion with O_2_-derived ligands bearing N-tetramethylated cyclam (TMC) chelates [1,3,4,5,6,7,8,9]. The TMC ligands are intensively used in biomimetic inorganic chemistry [1,3,4,10] due to their ability to modulate the binding mode of the O_2_ to the metal center and to influence its preferred oxidation state. 

The new resulting bioinspired complexes mimic the nitric oxide dioxygenases (NODs) which convert toxic nitric oxide (NO) to biologically benign NO_3_^−^ ion [10]. NODs play an essential biological role because, despite NO being a major signaling molecule in neurons and in the immune system, its overproduction can increase the formation of reactive nitrogen species implicated in different toxicological processes. The metal-superoxo assisted reaction usually proceeds via formation of peroxynitrite (-O_2_N=O), which is a species characterized by a short lifetime and involved in the production of NO_2_. Peroxynitrite, by fast recombination with the M-O core, generates NO_3_^−^ ion. For the process in which NO reacts with metal–peroxo species, information is more scarce.

In the last decade, some complexes involving a Cr(III) ion coordinated at two n-TMC ligands with increasing ring size, namely [Cr(III)(12-TMC)(O_2_)(Cl)]^+^ [10] and [Cr(III)(14-TMC)(O_2_)(Cl)]^+^, [5,11,12] have been proved to catalyze the oxidation of NO by O_2_ [5,10]. 

**Scheme 1:**
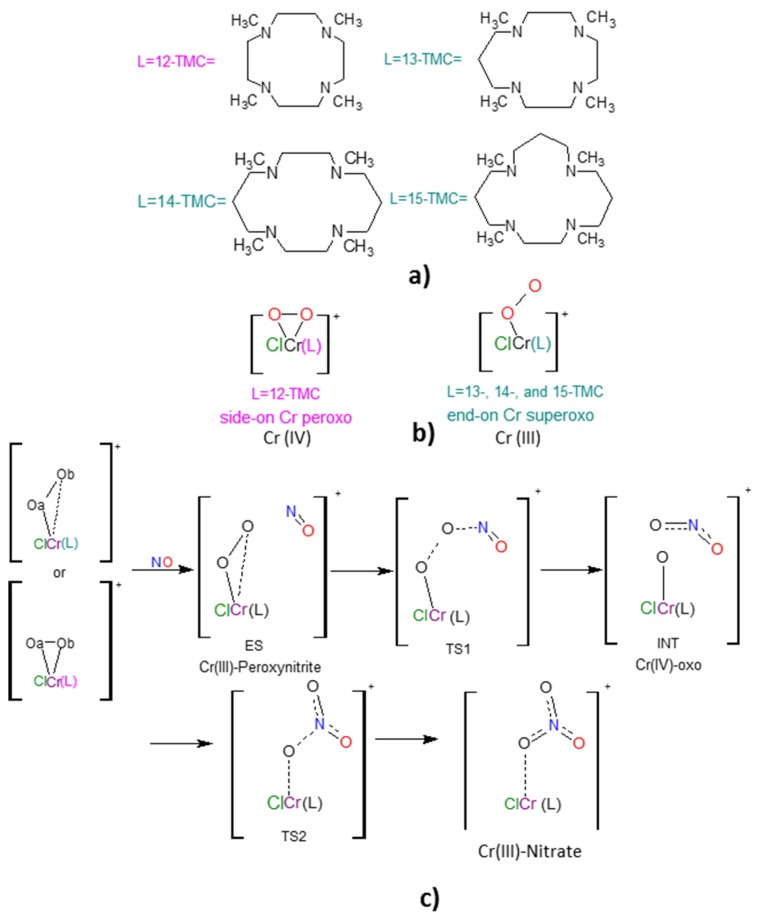
The N-tetramethylated cyclam (TMC) chelates of different ring size (**a**); the two different topologies (side-on peroxo and end-on superoxo) adopted for their complexes with O_2_ (**b**); and the considered mechanism for the conversion reaction of NO to NO_3_^−^ ion (**c**).

In order to give further insights on the reaction mechanism at atomistic level, we undertook a systematic theoretical study on all the synthetized complexes [10,11,12,13] (Scheme 1a), starting from the proposed mechanism [10] depicted in Scheme 1c. We will explain it in more detail later, as well as considering the possible ring size ligand effect ((n-TMC) with *n* = 12, 13, 14 and 15) on the energetics of the process.

## 2. Results and Discussion

### 2.1. Structural Aspects

As a first step of the work, we have optimized the initial [Cr(III)(n-TMC)O_2_(Cl)]^+^ and [Cr(IV)(n-TMC)O_2_(Cl)]^+^ (*n* = 12, 13, 14 and 15) complexes starting from the crystallographic structures where available [10,11,12,13]. The availability of crystallographic structures contributes to decrease the calculation time and allows comparison between experimental and theoretical parameters. Different spin multiplicities values (2S + 1 = 3, 5, 7) deriving from the two side-on (η2) and end-on (η1) binding modes of O_2_ molecule (Scheme 1b), which as mentioned before, determine the oxidation state of metal center in the complexes, have been considered. 

In the [Cr(III)(12-TMC)(O_2_)(Cl)]^+^ complex, the η2 side-on coordination of oxygen to the metal center is the preferred one, regardless of the electronic spin state. All the attempts to obtain the end-on η1 isomer failed since the structure collapses into the η2 one during the optimization. Support to these findings arises from the second-order perturbative analysis that reveals the presence of electronic delocalization in the Cr-O_2_ region involving the O-O bonding orbitals with the empty orbitals of the metal ion. The most stable spin configuration for the complex is the triplet electronic state followed by the quintet at 4.4 kcal mol^−1^. As expected, the state with multiplicity seven appears to be unstable since the oxygen molecule lies out from the metal coordination shell (Cr-O distances are 4.484 and 4.925 Å). The geometrical features of the minimum well match with the X-ray structure of a similar complex in which the chlorine atom is replaced by an aqua ligand [10].

For [Cr(III)(14-TMC)(O_2_)(Cl)]^+^ and [Cr(III)(15-TMC)(O_2_)(Cl)]^+^ global minima, our computations indicate the η1 coordination for oxygen and the triplet spin state. The excited quintet state lies at 5.5 and 6.2 kcal mol^−1^, respectively, above the absolute one. The end-on coordination of O_2_ as preferred binding mode in the [Cr(III)(14-TMC)(O_2_)(Cl)]^+^ complex, has already been suggested by the previous theoretical calculations performed at DFT/ B3LYP level of theory and by crystallographic studies [5,11,13]. The situation is different for the [Cr(III)(13-TMC)(O_2_)(Cl)]^+^ complex for which we still find the η1 as O_2_ preferred coordination mode but a ground state of quintet followed by the triplet lying at only 2.4 kcal mol^−1^.

The main geometrical parameters for the obtained minima of the considered species, in the most stable spin state and binding modes of O_2_, are summarized in Table 1.

As can be seen from Table 1, the Cr-Oa distance and the O-O-Cr valence angle increases in going from the complex involving 12-TMC to that with 14-TMC, but decreases with the 15-TMC.

This behavior can be explained by the different conformational flexibility of the ligands imposed by different rearrangements. In fact, in the complex [Cr(III)(15-TMC)(O_2_)(Cl)]^+^, the four methyl groups attached to the nitrogen atoms are equally distributed up and down the ring plane, while in the [Cr(III)(14-TMC)(O_2_)(Cl)]^+^, the four N-CH_3_ groups present a syn-type orientation with respect to the oxygen. This means that the end-on coordinated O_2_ suffers minor steric hindrance.

In the complex involving 12-TMC ligand, the topology is different and the axial positions are occupied by the peroxo ligand and one nitrogen atom, while the four pseudo-equatorial positions include three nitrogen and the chlorine atoms.

### 2.2. NO Conversion Mechanism

**Figure 1:**
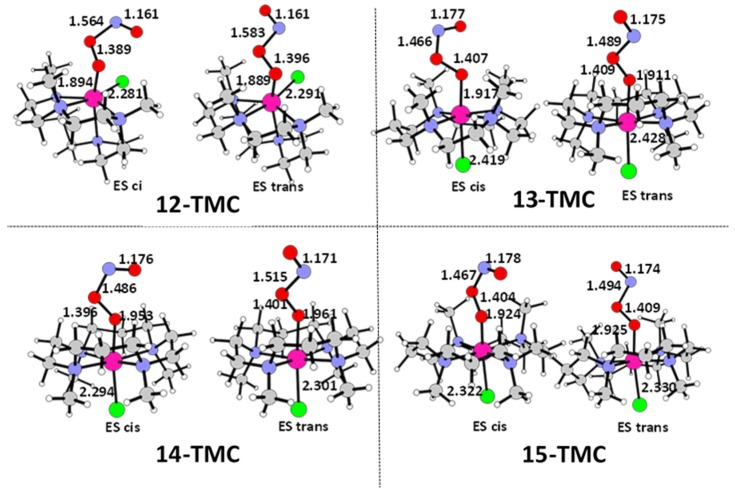
Optimized structures and main geometrical parameters of ES cis and trans peroxynitrite of the end-on [Cr(III)(n-TMC)(O_2_)(Cl)]^+^ (*n* = 13, 14 and 15) superoxo or side-on [Cr(IV)(n-TMC)(O_2_)(Cl)]^+^ (*n* = 12) peroxo complexes on NO. Selected bond lengths are in Å. Oxygen (red), chlorine (green), carbon (silver), nitrogen (azure), chromium (hot pink).

In the considered reaction mechanism, depicted in Scheme 1, the first step of the reaction is the nucleophilic attack of the end-on [Cr(III)(n-TMC)(O_2_)(Cl)]^+^ (*n* = 13, 14 and 15) superoxo or side-on [Cr(IV)(n-TMC)(O_2_)(Cl)]^+^ (*n* = 12) peroxo complexes on NO to form the peroxynitrite (ES) species. Since the peroxynitrite complexes can assume cis and trans isomeric forms, we have explored both these possibilities. We specify that, after the intermediate species (INT), the stationary point can assume only the cis arrangement. Combining the lowest lying electronic state of the peroxo or superoxo complexes with that of NO substrate, the ES complex can assume a spin multiplicity of doublet or quartet. For all the considered species, the quartet results to be the ground state, with the doublet being one that lies at higher energy (22.8, 23.2, 33.3, 24.2 kcal mol^−1^ for the peroxynitrite complexes with 12-TMC, 13-TMC, 14-TMC and 15-TMC ligands, respectively). Peroxynitrite species prefer the cis arrangement (Figure 1) and the trans isomers lie at about 4–5 kcal mol^−1^ above the cis ones. It is important to note that the formation of ES complexes occur without an energy barrier. The most stable species (ES cis peroxynitrite for 12-TMC, 13-TMC, 14-TMC and 15-TMC ligands) have been selected for its next conversion in NO_3_^−^ ion.

**Figure 2:**
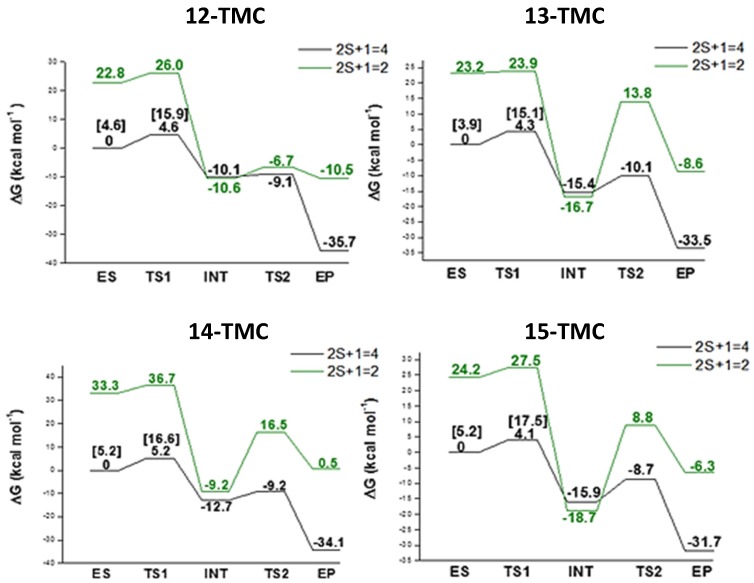
Potential energy surfaces for the NO oxidation reaction by end-on [Cr(III)(n-TMC)(O_2_)(Cl)]^+^ (*n* = 13, 14 and 15) superoxo and side-on [Cr(IV)(n-TMC)(O_2_)(Cl)]^+^ (*n* = 12) peroxo complexes at different spin states (doublet and quartet). The energy values in square brackets are referred to the trans isomer.

Following the proposed reaction mechanism of Scheme 1, for both the spin multiplicities, we have characterized all the minima and the transition states, whose cartesian coordinates are reported in the Supplementary Materials. The relative potential energy surfaces (PES) are reported in Figure 2. In the same figure, we also report the relative energies for the trans isomers in parenthesis.

From the peroxynitrite complex (ES) the formation of the NO_2_ species is obtained throughout the transition state TS1, in which the homolytic O-O cleavage and the N-O bond formation occur simultaneously. The potential energy surfaces concerning the doublet state lie at very higher energy with respect to those of quartet, therefore, we will discuss in detail only the second ones.

IRC computations clearly indicated that this transition state connects the ES with the intermediate INT. As it can be seen from Figure 2, the energy amount necessary to convert ES into INT is considerably small: 4.6 (12-TMC), 4.3 (13-TMC), 5.2 (14-TMC), and 4.1 (15-TMC) kcal mol^−1^. This means that with these activation energies (TS1), the first reaction step results to be very fast at laboratory temperature.

**Figure 3:**
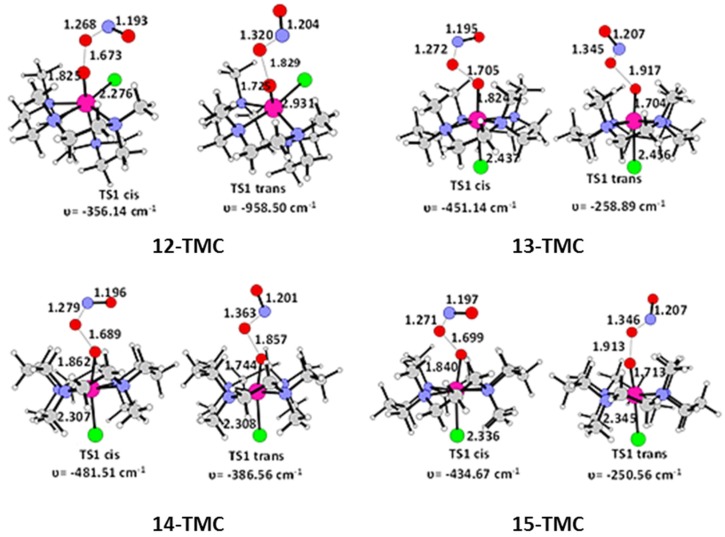
Optimized structures of cis and trans isomers of the first transition state (TS1) for all the investigated ligands along with the main geometrical parameters and imaginary frequency values. Selected bond lengths are in Å. Oxygen (red), chlorine (green), carbon (silver), nitrogen (azure), chromium (hot pink).

As it can be evinced from the Figure 3, where are depicted the TS1 optimized geometries in cis and in trans conformation of all complexes, the O-O distance reveals the breaking of the bond with the consequent formation of the O-N one that gives rise to the NO_2_ fragment.

As mentioned before, the quartet PESs wind below the doublet ones, but spin crossing at the first intermediate (INT) level are present on the PES describing the catalytic activity of complexes with 12-TMC, 13-TMC and 15-TMC. However, the energy values of intermediate in the two different multiplicities are very similar, therefore the inter-surface crossings should not introduce significant effects in the energetic behaviors.

**Figure 4:**
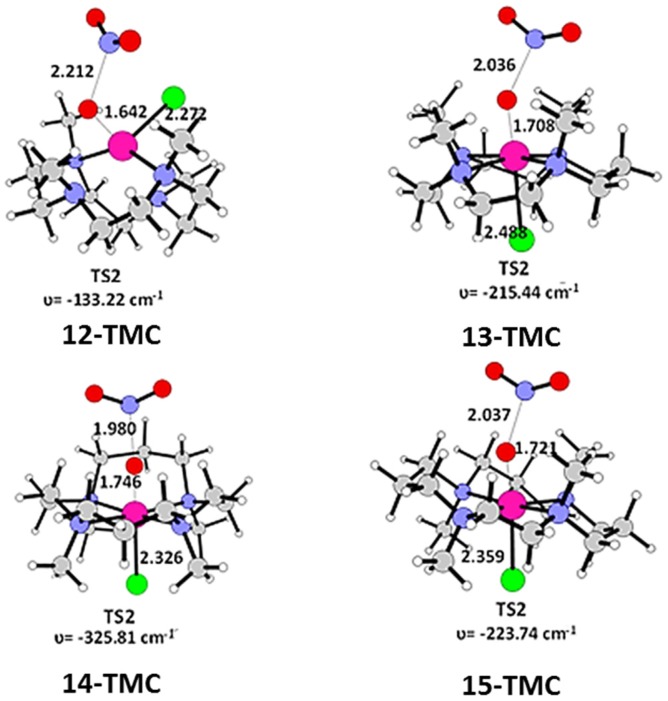
Optimized structures of TS2 species for all the investigated ligands along with the main geometrical parameters and imaginary frequency values. Selected bond lengths are in Å. Oxygen (red), chlorine (green), carbon (silver), nitrogen (azure), chromium (hot pink).

For the formation of the final product (EP), which implies the definitive conversion of NO_2_ in nitrate ion, the NO_2_ must approach the Cr=O moiety. In the PESs, it clearly shows that this event requires a small amount of energy. The highest barrier is found for the complex having 15-TMC (7.2 kcal mol^−1^, 10 kcal if the spin crossing is considered), while the lowest one is obtained for the complex with 12-TMC (1.0 kcal mol^−1^). Figure 4 shows, as in TS2, the NO_2_ is coordinated to Cr=O moiety and the new N-O bond is forming (N-O = 2.212, 2.036, 1.980 and 2.037 Å in 12, 13, 14 and 15-TMC, respectively).

**Figure 5:**
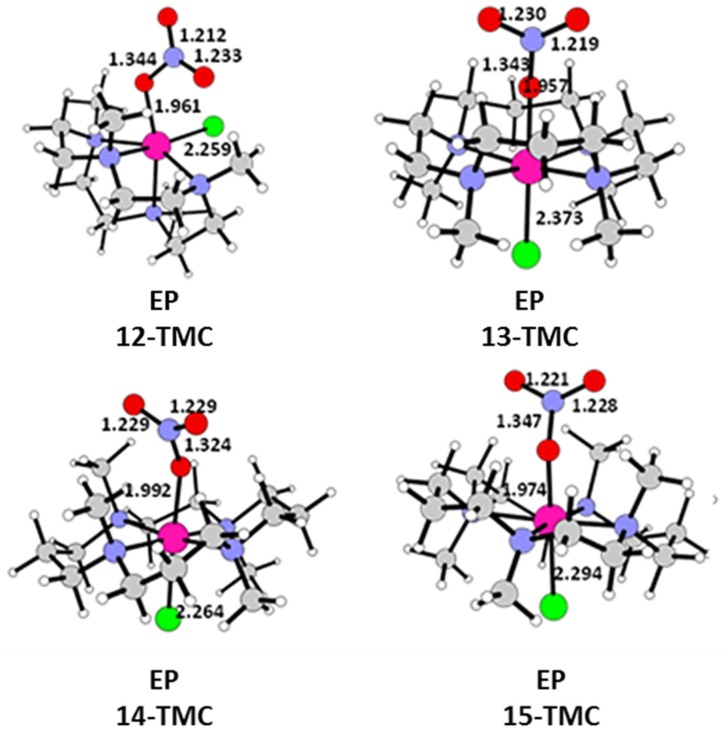
Optimized structures of EP species for all the investigated ligands along with the main geometrical parameters. Selected bond lengths are in Å. Oxygen (red), chlorine (green), carbon (silver), nitrogen (azure), chromium (hot pink).

In the final products (EP), the formed NO_3_^−^ is linked to the chromium ion throughout one oxygen (the distance is about 1.9 Å for all the considered cases), giving rise to a complex with a six-coordinated metal center (Figure 5). For the complex with 12-TMC ligand, our result concerning the Cr-O bond length (1.961 Å) can be compared with previous crystallographic X-ray data (2.003 Å).

The energetic potential energy surfaces we have determined allow to extrapolate some important information: (i) the formation of the peroxynitrite species occurs without an energy barrier and with a considerable energy stabilization; (ii) the nitrate ion formation process is strongly exothermic; (iii) the potential energy surfaces for the complex with different ligands are similar, indicating that all the starting complexes are able to easily oxidize NO in NO_3_^−^; and (iv) the spin inversion occurring along some reaction paths does not significantly affect the process.

**Figure 6:**
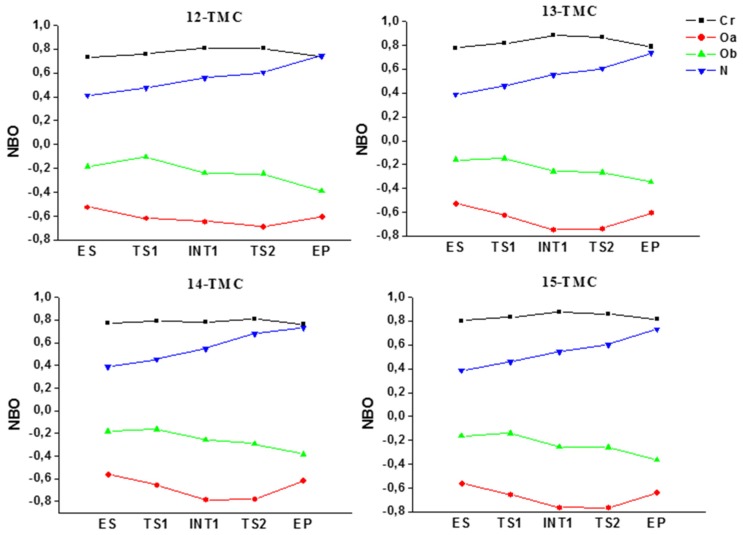
NBO charges in |e| for the atoms for every stationary point along the studied PESs (potential energy surfaces).

The NBO charges computed for the atoms for every stationary point along the PESs are reported in Figure 6. A common aspect to all examined cases is that the net charge of nitrogen atom increases in going from ES to EP indicating its activation. The negative charge of oxygen atom Oa linked to the metal center in the initial complex increases until the TS2 is reached, then it decreases in the nitrate final product due to the electronic delocalization on the NO_3_^−^.

## 3. Materials and Methods

All calculations have been performed using the density functional theory and employing the M06L [14,15] exchange-correlation functional as implemented in Gaussian 09 code [16]. This functional has been proven to give reliable results in describing electronic structure and properties of metal containing systems in various spin states [14,15]. Geometry optimizations have been performed using the 6-31+G(d,p) basis set for all atoms, except for chromium ion whose core and valence electrons have been described by SDD pseudopotential and the related basis set, respectively [17]. For each optimized stationary point, vibrational analysis has been performed for determining its minimum or saddle point character. The IRC method [18,19] has been employed to assess that the localized TSs correctly connect to the corresponding minima along the imaginary mode of vibration. To obtain more accurate electronic energies, single-point calculations at the M06L/6-311+G(2d,2p) level of theory and in solvent have been performed on the optimized geometries. The implicit solvent model SMD [20] has been used with a dielectric constant ε = 36.64 corresponding to the acetonitrile solvent employed in the experiment [10]. Spin contamination in terms of the <S2> values has been carefully examined in every intermediate and transition state in all the performed unrestricted calculations. The amount of spin contamination (S*2−S(S + 1)) is reported in Table 2 following the same procedure previously applied in chromium containing systems [21]. The geometry optimization was performed for each species under S = 3/2 spin state for Cr(III), and S = 0, 1 for Cr(IV)). The corresponding free energies were compared to determine the resting states of the complexes. 

Natural bond orbital (NBO) analysis has been carried out using the NBO 3.1 package [22] as implemented in Gaussian 09.

## 4. Conclusions

In summary, on the basis of obtained results, we can say that for the process catalyzed by side-on peroxo [Cr(IV)(12-TMC)(O_2_)(Cl)]^+^ and end-on superoxo [Cr(III)(14-TMC)(O_2_)(Cl)]^+^ complexes, the rate determining step is the NO_2_ formation. Reaction barriers are 4.1 and 5.2 kcal/mol, respectively. When end-on [Cr(III)(13-TMC)(O_2_)(Cl)]^+^ and [Cr(III)(15-TMC)(O_2_)(Cl)]^+^ superoxo catalysts are used to convert NO_2_ to NO_3_^−^, the biggest obstacle is the formation of the product. The energetic barriers are higher than those computed for the first two discussed cases but are, however, very low. In particular, for the reaction catalyzed by [Cr(III)(13-TMC)(O_2_)(Cl)]^+^, we have values of 5.3 kcal mol^−1^ and 7.2 kcal mol^−1^ that raises to 6.8 and 10.0 kcal mol^−1^, respectively, if spin inversions are considered. These values suggest that, at room temperature, the reactions should be very fast. From literature, we learned that only for the reaction catalyzed by the [Cr(III)(14-TMC)(O_2_)(Cl)]^+^ complex, it was possible to isolate the Cr(IV)-oxo (INT) intermediate. This might find an explanation in the fact that our data give, in this case, the highest barrier for (ES) peroxy→ Cr(IV)-oxo (INT) interconversion. In fact, a difference of about 1 kcal mol^−1^ between the barriers usually results in a reaction rate 10 times slower. Until now, experimental information is limited to the [Cr(III)(12-TMC)(O_2_)(Cl)]^+^ complex for which the EP has been isolated and characterized and the existence of a Cr(IV)-oxo intermediate has been hypothesized [10].

The low activation energy values determined by our calculations and hence, the very fast associated processes, might be the reason for the difficulty found in the experiments to isolate the reaction intermediates, although their existence has been postulated [5,10,11,13].

Based on what has been said, our computation can support the possibility to isolate the Cr(III)-nitrate (EP) species for the reactions catalyzed by [Cr(III)(13-TMC)(O_2_)(Cl)]^+^ and mainly [Cr(III)(15-TMC)(O_2_)(Cl)]^+^ since the barriers result to be the highest in the studied series (5.3 or 6.8 kcal mol^−1^ and 7.2 or 10.0 kcal mol^−1^, respectively). We hope that these results could stimulate future desirable experiments on the reactivity of chromium containing 13 and 15-TMC ligands.

## Figures and Tables

**Table 1:** Main geometrical parameters for the most stable structures of [Cr(III)(12-TMC)(O_2_)(Cl)]^+^, [Cr(III)(13-TMC)(O_2_)(Cl)]^+^, [Cr(III)(14-TMC)(O_2_)(Cl)]^+^ and [Cr(III)(15-TMC)(O_2_)(Cl)]^+^ complexes at M06L level of theory. Distances are in Å and valence angles are in degrees (°). See Scheme 1 for the labelled atoms.

	12-TMC	13-TMC	14-TMC	15-TMC
Cr-Cl	2.277	2.424	2.311	2.347
Cr-Oa	1.887	1.972	1.919	1.894
Cr-Ob	2.598	2.716	2.999	2.902
O-O	1.299	1.315	1.307	1.304
Cr-O-O	107.8	109.9	135.9	129.4

**Table 2:** Calculated spin contamination (S*2−S(S + 1)) for all investigated complexes under quartet spin state. TMC indicates the differently sized (12, 13, 14 and 15) N-tetramethylated cyclam chelates.

	12-TMC	13-TMC	14-TMC	15-TMC
ES_trans	3.7502	3.7502	3.7502	3.7502
ES_cis	3.7502	3.7502	3.7502	3.7502
TS1_trans	3.7502	3.7524	3.7502	3.7502
TS1_cis	3.7507	3.7507	3.7506	3.7507
INT	3.7519	3.8107	3.7519	3.7520

## Supplementary Materials

Supplementary materials can be found at https://www.mdpi.com/1422-0067/20/16/3955/s1.

## Abbreviations

TMC	N-tetramethylated cyclam I
NODs	Nitric oxide dioxygenases
PES	Potential Energy Surface
IRC	Intrinsic reaction coordinate
SMD	Solvation model based on the quantum mechanical charge density
NBO	Natural Bond Orbital
