# Supplementary material for: Computational Mechanistic Insights on the NO Oxidation Reaction Catalyzed by Non-Heme Biomimetic Cr-N-Tetramethylated Cyclam Complexes
Source: Int J Mol Sci. 2019 Aug 14;20(16):3955. doi: 10.3390/ijms20163955 (PMC6721035; doi:10.3390/ijms20163955)
Supplement: Supplementary file 1 [file ijms-20-03955-s001.pdf]

# Supporting Information

# Computational mechanistic insight on the NO oxidation reaction catalyzed by non-heme biomimetic Cr- N-tetramethylated cyclam complexes

*Tiziana Marino*, Maria Grazia Fortino, Nino Russo*, Marirosa Toscano, Marta Erminia Alberto*

*1 Dipartimento di Chimica e Tecnologie Chimiche, Università della Calabria, 87036 Rende, Italy;*

**Correspondence: tmarino@unical.it; nrusso@unical.it*

*Received: date; Accepted: date; Published: date*

| 12-TMC | | |
|---|---|---|
| **ES cis** | **ES trans** | **TScis** |
| Cr -0.14293200 -0.02131700 -0.11843500 | Cr 0.06313400 -0.14142200 -0.17768100 | Cr -0.12916600 -0.09513100 -0.09109400 |
| O -2.62124600 -1.02581300 0.51983400 | O 2.51822100 -0.94577700 0.41159400 | O -2.80288800 -1.38077900 0.32327300 |
| O -1.85619400 0.12336600 0.67490900 | O 1.28805800 -1.54550200 0.13398500 | O -1.66225700 -0.29070800 0.87857100 |
| C -1.12636200 2.80913100 -0.50877000 | C -0.92076200 -2.63924100 -1.54646500 | C -1.51176700 2.56313800 -0.50099100 |
| H -2.06583800 2.40203500 -0.13179700 | H 0.06325900 -3.02072900 -1.26996400 | H -2.34802100 2.03538400 -0.03822000 |
| H -1.08221300 2.61949100 -1.58295000 | H -0.82265100 -2.10264300 -2.49190700 | H -1.52171400 2.34000600 -1.56956300 |
| H -1.07625800 3.88813900 -0.31810700 | H -1.63013100 -3.46798100 -1.65894300 | H -1.61166200 3.64472800 -0.34593400 |
| N 1.83047500 0.36828000 -1.18796700 | N -1.70197700 1.11156600 -0.90876800 | N 1.69286000 0.51436800 -1.32857900 |
| N 0.00015200 2.10568300 0.15424500 | N -1.37864300 -1.69418100 -0.49775000 | N -0.24769200 2.06002400 0.09002000 |
| N 0.52562600 -0.02675000 2.00115800 | N -0.50212100 -0.46203700 1.93982200 | N 0.69845600 0.12870900 1.99554600 |
| N 0.71010000 -2.02442800 0.00310000 | N 0.62972100 1.81024800 0.67181300 | N 0.98263600 -1.92544600 0.04592600 |
| C 1.71985400 0.69071600 -2.63273700 | C -1.77388600 1.29458900 -2.37946000 | C 1.44279100 0.74919100 -2.77234400 |
| H 2.69426300 1.03799200 -3.00148300 | H -2.74010200 1.74522900 -2.64338700 | H 2.33568000 1.20195300 -3.22374600 |
| H 0.96009800 1.45165900 -2.80618100 | H -1.65140400 0.34295400 -2.89598800 | H 0.57880800 1.39680100 -2.91702100 |
| H 1.40540800 -0.19053000 -3.18751400 | H -0.95964700 1.93382100 -2.71521700 | H 1.21382600 -0.19133500 -3.26937000 |
| C 2.37826500 1.54366000 -0.48314100 | C -2.87767800 0.33754800 -0.46924100 | C 2.12715700 1.78454700 -0.71657700 |
| H 3.25048900 1.95775000 -1.01090100 | H -3.80612700 0.73072000 -0.91051200 | H 2.89516400 2.28273100 -1.32715800 |
| H 2.73642500 1.21914300 0.49633500 | H -2.97705800 0.45511600 0.61190900 | H 2.59654700 1.55548200 0.24249100 |
| C 1.30732200 2.61736100 -0.37161000 | C -2.71386000 -1.11981900 -0.86835400 | C 0.93195500 2.71370500 -0.55943100 |
| H 1.11309200 3.03759600 -1.36152800 | H -2.81599800 -1.21376600 -1.95194900 | H 0.60959800 3.05863000 -1.54505600 |
| H 1.66238500 3.44773100 0.25218000 | H -3.51558200 -1.72719800 -0.42943600 | H 1.22092400 3.61219800 0.00134300 |
| C -0.10880500 2.29981200 1.63031900 | C -1.43976000 -2.41212500 0.81040800 | C -0.27040800 2.31766300 1.55687800 |
| H -1.14804200 2.08070900 1.89538700 | H -0.49288200 -2.95125200 0.91423300 | H -1.24088400 1.96071000 1.91779300 |
| H 0.08636900 3.35054700 1.88490400 | H -2.24935100 -3.15418000 0.79124200 | H -0.21748100 3.39824200 1.74996900 |
| C 0.82348200 1.37750300 2.38851500 | C -1.61602800 -1.44830000 1.96470100 | C 0.84360200 1.58236500 2.27071800 |
| H 1.87260500 1.60006100 2.17741800 | H -2.57117400 -0.91953500 1.90598900 | H 1.82908200 1.92208100 1.94324600 |
| H 0.69262100 1.51530700 3.46901500 | H -1.62483600 -1.99543000 2.91576400 | H 0.79471100 1.77755700 3.34933200 |
| C -0.53414700 -0.55181400 2.90253600 | C 0.63536500 -1.01322200 2.72107900 | C -0.18780400 -0.45004000 3.03799900 |
| H -0.09820100 -0.75773200 3.88822200 | H 0.38528800 -0.99186500 3.78914100 | H 0.34068600 -0.44425200 4.00015300 |
| H -0.98187800 -1.45780600 2.49305800 | H 1.54393400 -0.43894300 2.53839100 | H -0.48405600 -1.46502200 2.77805300 |
| H -1.33770900 0.17487100 2.99433400 | H 0.84378900 -2.03230300 2.40307700 | H -1.10462100 0.13089500 3.11096100 |
| C 1.68868000 -0.93172900 2.05870900 | C -0.85716800 0.86436200 2.48311500 | C 1.96753300 -0.62498900 1.98101600 |
| H 1.97289300 -1.15047400 3.09854300 | H -0.95942300 0.82961700 3.57773000 | H 2.35912900 -0.76657300 2.99931200 |
| H 2.53942000 -0.41866100 1.60613300 | H -1.83836300 1.13975000 2.09102700 | H 2.71137500 -0.03142600 1.44543500 |
| C 1.35190000 -2.23084600 1.34399100 | C 0.21674000 1.87504600 2.11149500 | C 1.74274900 -1.98559100 1.33887400 |
| H 2.24934300 -2.85363300 1.23813800 | H -0.11514100 2.89088700 2.36336000 | H 2.70116100 -2.49739400 1.18413500 |
| H 0.65043300 -2.80641500 1.95291100 | H 1.11015000 1.68822700 2.71204900 | H 1.16630700 -2.61438700 2.02154800 |
| C -0.28211600 -3.09950300 -0.24283900 | C 2.07325600 2.13494700 0.56085100 | C 0.10014600 -3.11343600 -0.06779100 |
| H -0.74625000 -2.93503000 -1.21629200 | H 2.37232200 2.06463100 -0.48642700 | H -0.45487900 -3.05425500 -1.00538100 |
| H -1.06296700 -3.05160300 0.51744100 | H 2.65382700 1.41063100 1.13310200 | H -0.61575800 -3.10895600 0.75626700 |
| H 0.20093900 -4.08419100 -0.21771400 | H 2.26910000 3.14728400 0.93612400 | H 0.68815800 -4.03893100 -0.03818700 |
| C 1.72874400 -2.06544200 -1.08896500 | C -0.12262800 2.80354600 -0.15050500 | C 1.91440900 -1.90374600 -1.12227300 |
| H 1.17035100 -2.07862600 -2.03115500 | H 0.34567900 2.79893900 -1.14073000 | H 1.29475800 -2.03442800 -2.01610000 |
| H 2.30214700 -3.00073600 -1.02330600 | H 0.00307900 3.80790000 0.27813700 | H 2.60396000 -2.75716500 -1.06153700 |
| C 2.64807100 -0.86431800 -1.05317100 | C -1.58745400 2.45034400 -0.27858200 | C 2.67322800 -0.59609600 -1.20830100 |
| H 3.22581200 -0.82545000 -0.12674500 | H -2.08861400 2.44455900 0.69267600 | H 3.30533400 -0.43822300 -0.33098700 |
| H 3.38234700 -0.93269400 -1.86578900 | H -2.10282000 3.20870500 -0.88182400 | H 3.35030400 -0.61209000 -2.07164700 |
| Cl -1.06561400 -0.36714100 -2.17538900 | Cl 1.07871700 0.28063700 -2.18751300 | Cl -1.14666100 -0.60617500 -2.06165000 |
| O -3.89423100 0.39804900 -0.71024600 | O 4.43392600 -0.53682100 -0.66358700 | O -4.03190600 0.28094400 -0.35219100 |
| N -3.86896100 -0.71113600 -0.36932200 | N 3.45288200 -1.12861000 -0.85308900 | N -3.85031200 -0.88776200 -0.19287200 |
| **TS1trans** | **INT1** | **TS2** |
| Cr 0.11174200 -0.07920600 -0.13006600 | Cr 0.02968300 -0.01504800 -0.46843700 | Cr -0.21773000 -0.02655300 -0.09259900 |
| O 3.10103900 -0.05780800 0.48710600 | O -4.05742600 -1.01473300 -0.41259700 | Cl -1.34664200 0.31410200 -2.03488300 |
| O 1.47364700 -0.88943300 0.55103600 | O -1.44189000 0.51266000 -0.76316200 | N 1.77423100 0.34215300 -1.31581900 |
| C -0.11970100 -2.95082200 -1.03955600 | C 0.70600800 2.60453900 -1.70091300 | N 0.76771300 -0.10407100 2.00240100 |
| H 0.89733400 -3.00953000 -0.65004400 | H -0.36151700 2.58539100 -1.92339100 | N 0.81439700 -2.06080300 -0.14073000 |
| H -0.06685600 -2.58141900 -2.06499600 | H 1.22658000 2.03403000 -2.47226700 | N 0.09379100 2.06128600 0.27086100 |
| H -0.58970500 -3.94175500 -1.02598000 | H 1.06629100 3.64058600 -1.69980400 | N -3.55157500 -0.44586100 0.04088200 |
| N -1.87124600 0.50711800 -1.17342500 | N 2.41471300 -0.43813000 -0.03555700 | C -0.29345400 -0.09858500 3.03094600 |
| N -0.89894900 -1.99271600 -0.21664500 | N 0.94030000 1.97075500 -0.37672000 | H -0.83592500 -1.04203600 3.00411300 |
| N -0.61933600 -0.18470100 2.00780200 | N -0.51221900 0.64867900 1.74745700 | H -1.02564600 0.68451000 2.83102400 |
| N 0.05875300 2.01988100 0.34897500 | N 0.00249500 -1.90131000 0.56100700 | H 0.14907500 0.04859900 4.02492200 |

| | | |
|---|---|---|
| C -1.86444700 0.42365500 -2.65564400<br>H -2.88701600 0.56438600 -3.03119600<br>H -1.46981400 -0.53390100 -2.99438500<br>H -1.21252900 1.19108100 -3.06903300<br>C -2.85429300 -0.45983000 -0.65173500<br>H -3.80355500 -0.40996500 -1.20661900<br>H -3.08567200 -0.18863800 0.38050600<br>C -2.29106200 -1.86630000 -0.75920200<br>H -2.25601200 -2.16414900 -1.80954000<br>H -2.95300800 -2.58435000 -0.25807300<br>C -0.91233300 -2.46745300 1.19912800<br>H 0.12447000 -2.70395500 1.45833800<br>H -1.49976700 -3.39285300 1.27422000<br>C -1.45057800 -1.40859300 2.13882200<br>H -2.49360500 -1.16938200 1.91925500<br>H -1.43330100 -1.78006000 3.17116800<br>C 0.51706400 -0.28131000 2.95649800<br>H 0.14813400 -0.13069200 3.97889900<br>H 1.28619000 0.45397600 2.72341100<br>H 0.98977600 -1.25674300 2.87257900<br>C -1.33895800 1.08073700 2.24076300<br>H -1.55086800 1.23379400 3.30956000<br>H -2.30792400 1.02384400 1.74045000<br>C -0.49131800 2.23507900 1.73202600<br>H -1.05812200 3.17421200 1.76287400<br>H 0.36245500 2.37150800 2.39867600<br>C 1.40047400 2.64959900 0.24993600<br>H 1.82139500 2.43856400 -0.73422200<br>H 2.05935300 2.21996400 1.00444500<br>H 1.32714600 3.73333000 0.40123900<br>C -0.82799500 2.64686700 -0.67934300<br>H -0.28287400 2.59891600 -1.62787200<br>H -0.98178400 3.70583200 -0.43004200<br>C -2.14644900 1.91852100 -0.80425500<br>H -2.71867100 1.95822500 0.12551200<br>H -2.77047500 2.40571000 -1.56454000<br>Cl 1.10827700 0.19882200 -2.14534000<br>O 4.92010500 -0.40590100 -0.55494100<br>N 3.78821100 -0.77825900 -0.37991400 | C 3.24945000 -0.63915300 -1.23900300<br>H 4.31184300 -0.65405200 -0.95832200<br>H 3.07045700 0.14549500 -1.97457700<br>H 2.98730300 -1.57904500 -1.72270200<br>C 2.87331700 0.76155400 0.67545600<br>H 3.97053400 0.79149900 0.76707500<br>H 2.47909500 0.72873400 1.69373400<br>C 2.40299100 1.99641300 -0.06627400<br>H 2.93588900 2.07110500 -1.01753300<br>H 2.64609400 2.90564000 0.49837700<br>C 0.15271100 2.71609000 0.64960900<br>H -0.85732000 2.82971300 0.24329900<br>H 0.57781400 3.72065500 0.78059400<br>C 0.09253000 1.97737200 1.96947100<br>H 1.08685300 1.86401100 2.40880000<br>H -0.49333900 2.56217700 2.69153900<br>C -1.97712800 0.75595800 1.87749100<br>H -2.25108800 0.94735900 2.92375400<br>H -2.46635400 -0.15556100 1.53099300<br>H -2.34585600 1.56572900 1.24838800<br>C 0.00239700 -0.41717500 2.61440600<br>H -0.41706200 -0.35999000 3.63096400<br>H 1.08161900 -0.28273200 2.71831400<br>C -0.34037300 -1.77083000 2.01699400<br>H 0.14909700 -2.57456100 2.58077400<br>H -1.41579600 -1.94308400 2.10631300<br>C -1.03255700 -2.74610700 -0.09976900<br>H -0.78552000 -2.85101100 -1.15695800<br>H -2.00635800 -2.26214000 -0.01384500<br>H -1.07042800 -3.73505700 0.37237600<br>C 1.32626000 -2.56686000 0.36896800<br>H 1.40508900 -2.79559600 -0.69910100<br>H 1.33694900 -3.51589900 0.92192600<br>C 2.46624500 -1.66859800 0.78365200<br>H 2.40506600 -1.41127000 1.84448700<br>H 3.42244900 -2.19293800 0.65346700<br>Cl 0.49152300 -0.85554100 -2.50369500<br>O -4.43020700 1.13570500 -0.74884600<br>N -4.39551500 -0.03172300 -1.02119600 | C 0.92293900 2.57851100 -0.86090800<br>H 1.17044400 3.63333400 -0.67923000<br>H 0.29109700 2.52862800 -1.75411000<br>C 1.56962400 1.12417500 2.10568100<br>H 2.49138600 0.98037900 1.53771900<br>H 1.87471700 1.32411300 3.14437800<br>C 1.54924400 -1.35854000 2.12848500<br>H 1.59576100 -1.67949000 3.17739500<br>H 2.58251400 -1.17697800 1.82206900<br>C -0.08666200 -2.99881600 -0.84322500<br>H -0.20014400 -2.68034900 -1.88208100<br>H -1.07017900 -2.96353800 -0.37057100<br>H 0.30245600 -4.02494900 -0.81620900<br>C 2.76053200 -0.63633200 -0.82353500<br>H 3.08367600 -0.31981000 0.17038000<br>H 3.66272200 -0.64656500 -1.45468100<br>C 0.90574500 -2.44430600 1.28865900<br>H 1.45749600 -3.38948100 1.39815300<br>H -0.11992300 -2.61507100 1.63244100<br>C 1.61027200 0.15638300 -2.77642100<br>H 0.97665900 0.94218800 -3.18408500<br>H 1.11203400 -0.78900700 -2.99281100<br>H 2.59376700 0.18004100 -3.26543400<br>C -1.20922600 2.77852900 0.25703200<br>H -1.05717200 3.84940700 0.43840900<br>H -1.85864900 2.36570000 1.03127900<br>H -1.68779900 2.62954800 -0.71160400<br>C 0.76497400 2.30540700 1.59360700<br>H 1.39698500 3.19954300 1.52372000<br>H -0.02337100 2.54345800 2.31179900<br>C 2.14999200 -2.02699700 -0.79561700<br>H 2.83680200 -2.72943100 -0.30396800<br>H 2.02621900 -2.39063400 -1.81916700<br>C 2.16991500 1.74948900 -1.06384700<br>H 2.75293900 2.15067100 -1.90330600<br>H 2.82641900 1.79565500 -0.19069000<br>O -1.46241800 -0.74106400 0.70423100<br>O -3.98510100 -1.45758600 -0.41048000<br>O -3.97238200 0.64448500 0.28715300 |
| EP | | |
| Cr -0.07834900 -0.03230300 -0.28520700<br>Cl -0.22187200 0.41088000 -2.49543800<br>N 2.16961900 0.14912900 -0.41865900<br>N -0.32869500 -0.19504100 1.88587800<br>N 0.50779200 -2.11528300 -0.06651500<br>N 0.16792700 2.11156400 0.28163800<br>N -2.93996600 -0.13339300 -0.63643600<br>C -1.76030300 -0.03696800 2.26208200<br>H -2.31801600 -0.92543700 1.97209800<br>H -2.20207600 0.81675600 1.74582900<br>H -1.83712100 0.10379400 3.34692700<br>C 1.51046500 2.48265700 -0.25653200<br>H 1.78297900 3.48571300 0.10046400<br>H 1.39702100 2.53858400 -1.34486900<br>C 0.44645200 0.91396000 2.48610300<br>H 1.50498200 0.65586000 2.42125400<br>H 0.21637000 1.01545300 3.55656200<br>C 0.13217300 -1.54220900 2.32500000<br>H -0.39413100 -1.84509900 3.23826100<br>H 1.19250900 -1.49452900 2.58465300<br>C -0.01274100 -2.97005500 -1.16549800<br>H 0.38995000 -2.61393200 -2.11575300<br>H -1.09837800 -2.88887700 -1.19959100<br>H 0.28098300 -4.01451800 -1.00687300<br>C 2.69769500 -0.96939000 0.38998900<br>H 2.55134100 -0.72760900 1.44486100<br>H 3.78225800 -1.08084400 0.24612300 | | |

| | | |
|---|---|---|
| C -0.12799000 -2.54083300 1.21758400 | | |
| H 0.23465100 -3.53868600 1.49884100 | | |
| H -1.20095300 -2.61940000 1.02409100 | | |
| C 2.69327300 0.01477000 -1.80433200 | | |
| H 2.42543000 0.89105200 -2.39069400 | | |
| H 2.24708200 -0.84218800 -2.30712300 | | |
| H 3.78490100 -0.09210600 -1.76638800 | | |
| C -0.78774900 3.09203700 -0.29991700 | | |
| H -0.43786500 4.11257000 -0.10010800 | | |
| H -1.77444100 2.95159300 0.13278700 | | |
| H -0.85636200 2.92388600 -1.37580500 | | |
| C 0.12827600 2.21775600 1.77537000 | | |
| H 0.80605000 3.01069900 2.11904100 | | |
| H -0.88056900 2.53913500 2.04569100 | | |
| C 1.99875100 -2.25412000 -0.00495900 | | |
| H 2.27063700 -3.06764100 0.67926800 | | |
| H 2.33640900 -2.56214200 -0.99750800 | | |
| C 2.57852900 1.47990000 0.09919900 | | |
| H 3.53985100 1.78199600 -0.33477500 | | |
| H 2.73778300 1.42123300 1.17881200 | | |
| O -1.84918300 -0.86966000 -0.36474100 | | |
| O -3.95621700 -0.74355900 -0.88935900 | | |
| O -2.80045100 1.08961100 -0.57021700 | | |

| 13 TMC | | |
|---|---|---|
| ES cis | ES trans | TScis |
| O -1.14874800 -0.28748500 -1.27203900 | O 0.97607300 -0.27241600 -1.41540800 | O -1.07307900 -0.40576600 -1.20381400 |
| N -1.12496400 0.74103100 1.52380300 | O 2.22155800 0.38598000 -1.41115400 | N -1.11835700 0.37976200 1.64159400 |
| N -0.32575900 -1.91315700 0.86811500 | N -0.46485300 2.05156000 -0.32040300 | N 0.11367300 -2.01779900 0.70809000 |
| N 1.51074100 -1.15578800 -1.16146200 | N -1.81770800 -0.23817000 -1.34152700 | N 1.63191100 -0.77121900 -1.34371400 |
| N 1.18764600 1.63439000 -0.79571000 | N -0.38781700 -2.12678700 0.22076200 | N 0.81900700 1.87427600 -0.68909000 |
| C -1.11281500 -0.26424100 2.65545400 | N 1.11596700 -0.21150200 1.67588600 | C -0.86502000 -0.70576100 2.66626600 |
| H -2.14298300 -0.36840700 3.01061700 | C -1.90930800 2.23865100 -0.73194200 | H -1.84089600 -1.04405800 3.02840300 |
| H -0.53132400 0.16315400 3.47481400 | H -1.91903900 2.93389800 -1.57703400 | H -0.34766800 -0.25164700 3.51440700 |
| C -0.51380500 -1.65517400 2.33294000 | H -2.43496300 2.72733700 0.09147100 | C -0.02140000 -1.91409700 2.19627700 |
| H 0.45969100 -1.74035900 2.81029100 | C -2.69169000 0.95357800 -1.08912900 | H 0.97999500 -1.83270900 2.61185700 |
| H -1.16306500 -2.43875500 2.74351000 | H -3.36013500 0.70106200 -0.27000500 | H -0.47236600 -2.84164300 2.57165700 |
| C 0.83033000 -2.84157000 0.55398600 | H -3.30117700 1.13413100 -1.98378400 | C 1.40059000 -2.67817200 0.25963700 |
| H 0.41752600 -3.66583200 -0.03432600 | C -2.48618000 -1.55817500 -1.02185400 | H 1.11447500 -3.53061700 -0.36308800 |
| H 1.21003300 -3.27943900 1.48161500 | H -2.44215700 -2.16169500 -1.93278200 | H 1.92217000 -3.08801800 1.12937400 |
| C 1.99843000 -2.19530000 -0.20684100 | H -3.54598200 -1.38901100 -0.81069000 | C 2.36983700 -1.77115300 -0.51730100 |
| H 2.69484700 -1.71891400 0.47970500 | C -1.86457900 -2.33817300 0.14819300 | H 3.02677800 -1.22925400 0.15887200 |
| H 2.53658400 -2.97893600 -0.75890100 | H -2.29310700 -2.02453300 1.09766200 | H 2.99168200 -2.39964800 -1.17079700 |
| C 2.58632800 -0.23585100 -1.66853400 | H -2.07581100 -3.40825400 0.01086700 | C 2.47105600 0.38151400 -1.82075900 |
| H 2.41886900 -0.12425000 -2.74327600 | C 0.22446900 -2.53705100 1.53089000 | H 2.20418100 0.54535200 -2.86861400 |
| H 3.56969800 -0.70465700 -1.55306100 | H 1.13893900 -3.08623000 1.28959200 | H 3.53177200 0.10773800 -1.80892700 |
| C 2.58467000 1.13245400 -0.99598300 | H -0.43358100 -3.24169600 2.05095500 | C 2.26319700 1.66733300 -1.02687100 |
| H 3.06431900 1.09005900 -0.02007100 | C 0.54784500 -1.36223400 2.44854500 | H 2.82063800 1.64656100 -0.09254000 |
| H 3.14312700 1.84185500 -1.62213600 | H -0.34362400 -1.00828600 2.96185200 | H 2.62486400 2.51785600 -1.62133600 |
| C 0.65321900 2.07399500 -2.10510100 | H 1.27196800 -1.68908300 3.20748400 | C 0.09674500 2.30284000 -1.90818500 |
| H -0.32890000 2.52550600 -1.98323600 | C 2.51256700 -0.53741000 1.31320800 | H -0.93502800 2.55012400 -1.66656400 |
| H 0.54216900 1.21970400 -2.77517700 | H 2.99397700 0.32121700 0.84769100 | H 0.07762900 1.49454800 -2.64106400 |
| H 1.33652800 2.80528400 -2.55370500 | H 2.53551600 -1.36437800 0.59868800 | H 0.58607900 3.18480800 -2.33981900 |
| C 1.14986400 2.78351000 0.16442700 | H 3.07199200 -0.81996700 2.21332100 | C 0.65240400 2.91027300 0.37855600 |
| H 1.45642100 3.68789600 -0.37801200 | C 1.09999100 1.05711200 2.47405700 | H 0.75799600 3.89887200 -0.08831500 |
| H 1.89395500 2.59223000 0.94063400 | H 1.95505700 1.03592200 3.16282000 | H 1.47101400 2.78474300 1.09045600 |
| C -0.22433500 3.00609800 0.79489500 | H 0.18750700 1.05987900 3.07417200 | C -0.68811100 2.82824700 1.10725500 |
| H -0.22647400 4.02744000 1.18926000 | C 1.16739600 2.31659000 1.60978400 | H -0.83237200 3.79139000 1.60730600 |
| H -1.00238100 3.00601300 0.01941600 | H 1.49619600 3.13028400 2.26402000 | H -1.51247800 2.76604500 0.38346100 |
| C -0.62264900 2.09298500 1.95714600 | H 1.96611400 2.21869300 0.86123600 | C -0.83407800 1.75384500 2.18668500 |
| H 0.22748900 1.93885900 2.62337800 | C -0.13234800 2.78550100 0.94985700 | H 0.07740900 1.69657200 2.78358900 |
| H -1.42263200 2.57356800 2.53579600 | H -0.96885700 2.65798400 1.63853500 | H -1.66228200 2.02267800 2.85600400 |
| C 0.88442200 -1.83565600 -2.31978300 | H -0.05037300 3.85296900 0.70505100 | C 1.07303400 -1.46386800 -2.52819100 |
| H 0.43270500 -1.10174700 -2.98596300 | C 0.25779200 -2.90037600 -0.86649800 | H 0.43237000 -0.78521300 -3.08935100 |

| | | |
|---|---|---|
| H 0.09078100 -2.50291300 -1.98201400 | H 1.32607400 -2.68667700 -0.89698900 | H 0.46073700 -2.30964800 -2.21393000 |
| H 1.63953900 -2.42157700 -2.85777600 | H -0.15812300 -2.61062700 -1.83220800 | H 1.88964400 -1.82927300 -3.16279900 |
| C -1.57800700 -2.51339000 0.34232900 | H 0.08839600 -3.97265900 -0.70983500 | C -1.03803400 -2.79804100 0.19133000 |
| H -1.56130000 -2.55659800 -0.74633300 | C -1.43494100 -0.22827200 -2.77454000 | H -1.06409600 -2.77092900 -0.89783300 |
| H -2.43164100 -1.89902700 0.62883000 | H -0.69437600 -0.99985500 -2.98240600 | H -1.97110300 -2.36059500 0.54756700 |
| H -1.70949000 -3.51691800 0.76347200 | H -0.98914400 0.73355300 -3.02884900 | H -0.97349500 -3.83363100 0.54585500 |
| C -2.52737700 0.91827300 1.07049000 | H -2.32483400 -0.38384900 -3.39552200 | C -2.55368100 0.34350600 1.26795000 |
| H -2.90947200 -0.00712000 0.64060600 | C 0.40219700 2.60523000 -1.39193900 | H -2.78512500 -0.57712100 0.73231300 |
| H -2.58311000 1.69088300 0.30585500 | H 0.29761800 2.02328500 -2.30635600 | H -2.78929000 1.18403400 0.61710500 |
| H -3.15366800 1.21186100 1.92044900 | H 1.44798600 2.55848700 -1.09566800 | H -3.17324700 0.41360200 2.16924100 |
| Cr 0.19592200 -0.02800600 0.06637200 | H 0.12821700 3.64945300 -1.58142400 | C 0.20281900 -0.01259100 0.03829800 |
| Cl 1.84218100 0.11655700 1.82521000 | Cr -0.23486400 -0.02013900 0.04094600 | Cl 1.93529100 0.29084200 1.72580500 |
| N -3.26550500 0.41585500 -2.09476900 | Cl -1.94642900 0.31903500 1.72908400 | N -3.44468700 0.08929700 -1.87391500 |
| O -3.53072300 -0.69800500 -1.82110700 | N 3.26330700 -0.62250400 -1.74969500 | O -3.64837200 -1.07091200 -1.66997900 |
| O -1.88450600 0.79657500 -1.78537400 | O 4.30664700 -0.08476500 -1.80293200 | O -2.32200600 0.65392400 -1.67583100 |
| TS1trans | INT1 | TS2 |
| O 1.03001900 -0.31489300 -1.09842600 | O -1.39084800 -0.19321500 -0.13215500 | O 0.96967800 -0.39877000 -1.18800800 |
| O 2.78094800 0.43091900 -1.32833700 | N 0.08000500 1.11398400 1.75533000 | N -0.27970000 2.01839600 -0.55622100 |
| N -0.56593700 1.94525600 -0.64166700 | N 0.54713800 -1.65015900 1.27747900 | N -1.74854800 -0.27958700 -1.38001100 |
| N -1.66920400 -0.55516700 -1.45385200 | N 0.55258400 -1.47185000 -1.54111400 | N -0.50661000 -2.07957000 0.43000200 |
| N -0.28385500 -2.10002200 0.48089100 | N 0.42480800 1.37412200 -1.54709900 | N 0.97127600 -0.06154700 1.78131300 |
| N 0.84995400 0.14501300 1.80605400 | C 0.71989300 0.30840100 2.86573700 | C -1.65481300 2.25148800 -1.14561200 |
| C -1.93278700 1.96917300 -1.29309900 | H -0.02600000 0.21053200 3.66028300 | H -1.51245900 2.77786200 -2.09450800 |
| H -1.82289600 2.48647300 -2.25130500 | H 1.55009000 0.88318800 3.28613600 | H -2.20116900 2.92984700 -0.48539400 |
| H -2.59403300 2.58096000 -0.67471900 | C 1.25531600 -1.08237000 2.46994200 | C -2.53180300 0.99809900 -1.35772900 |
| C -2.62044000 0.60130200 -1.50034700 | H 2.31298600 -1.02591200 2.22839000 | H -3.26333600 0.92427000 -0.55817200 |
| H -3.36854700 0.44753500 -0.72763000 | H 1.12863500 -1.77082600 3.31593900 | H -3.07330100 1.09190800 -2.30801100 |
| H -3.12881600 0.59533800 -2.47317100 | C 1.34574600 -2.68407300 0.51186700 | C -2.53573900 -1.49217100 -0.92651500 |
| C -2.29474000 -1.85823000 -0.99932300 | H 0.79131000 -3.62522200 0.57842000 | H -2.55778500 -2.18708000 -1.77142100 |
| H -2.15658400 -2.57334400 -1.81551400 | H 2.30119200 -2.84115100 1.01936600 | H -3.57042700 -1.19647100 -0.73353600 |
| H -3.37276300 -1.71866400 -0.88191600 | C 1.62585400 -2.33315300 -0.96365500 | C -1.98931900 -2.20357300 0.32513400 |
| C -1.72708200 -2.43921600 0.30792300 | H 2.56776500 -1.79571300 -1.05455100 | H -2.42448200 -1.78012400 1.22808200 |
| H -2.26682900 -2.05147800 1.16913400 | H 1.70064600 -3.26365700 -1.54379100 | H -2.26386300 -3.26708100 0.27551000 |
| H -1.84547100 -3.53173800 0.29015100 | C 0.95263400 -0.72828500 -2.78393900 | C 0.03305300 -2.36805600 1.79976900 |
| C 0.22528900 -2.26686000 1.88378500 | H 0.09320900 -0.77338100 -3.45841700 | H 0.95818000 -2.93356400 1.65580700 |
| H 1.21913200 -2.71513700 1.80020700 | H 1.77277800 -1.24983200 -3.29022300 | H -0.65009100 -3.02667000 2.34816300 |
| H -0.39617200 -2.98548100 2.42972800 | C 1.34208200 0.72497600 -2.53703900 | C 0.30866100 -1.11355900 2.61778900 |
| C 0.30755600 -0.95935000 2.66192400 | H 2.35358400 0.79972500 -2.14018500 | H -0.61459700 -0.68796500 3.00815400 |
| H -0.67353900 -0.64925300 3.01715800 | H 1.30364100 1.27504500 -3.48735400 | H 0.95719800 -1.36959200 3.46712400 |
| H 0.96116900 -1.09744900 3.53373900 | C -0.88990600 1.63325900 -2.18614200 | C 2.37697400 -0.45320600 1.54102800 |
| C 2.30448700 -0.06646100 1.61655900 | H -1.57739600 2.07022000 -1.46200600 | H 2.88939400 0.33147800 0.98461200 |
| H 2.73527700 0.76355500 1.05865200 | H -1.33306900 0.69570500 -2.52415700 | H 2.42436700 -1.37934300 0.96385600 |
| H 2.48284300 -0.97676900 1.04101500 | H -0.76191400 2.31433700 -3.03629000 | H 2.89322400 -0.60013700 2.49793900 |
| H 2.79546700 -0.14166700 2.59419700 | C 0.99525900 2.66221600 -1.04280700 | C 0.94956200 1.27625300 2.45583300 |
| C 0.62631900 1.48751400 2.43433500 | H 0.84964100 3.41919100 -1.82555900 | H 1.73714900 1.27829900 3.22141100 |
| H 1.38101000 1.61755200 3.22133600 | H 2.06886300 2.51612200 -0.91092700 | H -0.01311700 1.37413800 2.96087000 |
| H -0.35641900 1.47012100 2.91018600 | C 0.37105800 3.15494700 0.25979600 | C 1.16104500 2.44841300 1.49907700 |
| C 0.71383100 2.64840400 1.44607700 | H 0.69866000 4.19302000 0.37890400 | H 1.42443700 3.30959300 2.12190500 |
| H 0.82653700 3.55774200 2.04520000 | H -0.71987700 3.22249300 0.16575300 | H 2.04817300 2.28279400 0.87305300 |
| H 1.64211100 2.58693000 0.86246400 | C 0.76354300 2.43553400 1.54997900 | C -0.03175700 2.88337900 0.64829800 |
| C -0.48767600 2.88491400 0.53023600 | H 1.83959700 2.25917200 1.56931500 | H -0.93982200 2.88090700 1.25222600 |
| H -1.41529800 2.79019900 1.09597300 | H 0.50526200 3.07454600 2.40596000 | H 0.13787200 3.90789800 0.28984200 |
| H -0.43756200 3.90579600 0.12847400 | C -0.62496200 -2.31084800 -1.85726500 | C 0.12158900 -3.01047700 -0.53406900 |
| C 0.52492800 -2.95661600 -0.41900000 | H -1.47704700 -1.67660700 -2.10417100 | H 1.19388100 -2.82509500 -0.59054400 |
| H 1.56119300 -2.61726100 -0.42779900 | H -0.89608800 -2.91512000 -0.99115500 | H -0.29264600 -2.85075100 -1.53004100 |
| H 0.14597800 -2.88661700 -1.43922900 | H -0.39208100 -2.98294300 -2.69186900 | H -0.07276600 -4.04713100 -0.23270000 |
| H 0.46427000 -4.00130300 -0.09125600 | C -0.73448500 -2.24101800 1.74480400 | C -1.27480200 -0.49199900 -2.77039400 |
| C -1.09948700 -0.72750600 -2.81334100 | H -1.34896200 -2.54258600 0.89719600 | H -0.58448800 -1.33333400 -2.81484800 |
| H -0.29296100 -1.45977900 -2.80200000 | H -1.29675100 -1.49355900 2.30623800 | H -0.73706700 0.39420700 -3.10991700 |
| H -0.68151300 0.22001400 -3.15441400 | H -0.53458800 -3.10244400 2.39225200 | H -2.13087300 -0.66654000 -3.43261300 |
| H -1.88623800 -1.04184100 -3.50888600 | C -1.33517500 1.38342100 2.11941400 | C 0.73746600 2.36657300 -1.57927300 |
| C 0.44526000 2.38701800 -1.63884700 | H -1.86943600 0.44715800 2.27169100 | H 0.61678500 1.73568700 -2.45839400 |
| H 0.47399700 1.70062100 -2.48378900 | H -1.84141400 1.90706200 1.30828700 | H 1.73956500 2.19003500 -1.19155200 |
| H 1.43901000 2.38657000 -1.19311600 | H -1.37414900 1.98744300 3.03400400 | H 0.63293800 3.42168800 -1.86011200 |
| H 0.20014800 3.39793000 -1.98494800 | Cr 0.22593200 -0.04620500 -0.00951200 | Cr -0.18580300 -0.01965900 0.01186400 |
| Cr -0.23489800 -0.02938700 0.00771700 | Cl 2.69504500 0.29648600 0.23670400 | Cl -2.03790100 0.62132900 1.54383600 |
| Cl -2.19423900 0.38643200 1.42967700 | N -4.08925600 -0.43362800 -0.33824100 | N 2.98684500 -0.48036900 -1.45406100 |

| | | |
|---|---|---|
| N 3.56262000 -0.66297300 -1.31585300<br>O 4.70645100 -0.31013600 -1.47423100 | O -4.12090400 -0.66010900 0.84215000<br>O -4.10766200 0.57674000 -0.99022000 | O 3.28991600 -1.57814200 -1.81142000<br>O 3.53655300 0.58456600 -1.46231100 |
| EP | | |
| O 0.61457100 -0.46097800 1.62307700<br>N 1.36449000 -0.80120700 -1.31732400<br>N -1.01759500 -1.89193700 -0.22938300<br>N -1.92154400 0.52571800 0.97214700<br>N 0.20735800 2.11535700 0.03489900<br>C 0.67861800 -1.86792300 -2.14269200<br>H 1.37268700 -2.70898300 -2.23365100<br>H 0.52537400 -1.46458300 -3.14515700<br>C -0.69205500 -2.35828800 -1.61832900<br>H -1.47456000 -1.99025800 -2.27871600<br>H -0.72571000 -3.45452400 -1.63239700<br>C -2.50075300 -1.71415300 0.01824800<br>H -2.74256400 -2.31298000 0.90059900<br>H -3.06667200 -2.14378200 -0.81353600<br>C -2.95788000 -0.26612100 0.23946600<br>H -3.16008800 0.23605500 -0.70400500<br>H -3.88815400 -0.27709800 0.82466900<br>C -2.13120400 2.01604600 0.89732500<br>H -2.02104300 2.39836600 1.91552000<br>H -3.16025700 2.23320800 0.59276000<br>C -1.15544400 2.72960000 -0.03571200<br>H -1.49215500 2.68207700 -1.06861200<br>H -1.09296700 3.78781300 0.25197800<br>C 0.84355500 2.53606300 1.30626300<br>H 1.85492600 2.14198100 1.37181200<br>H 0.28004700 2.14313100 2.15533900<br>H 0.86288100 3.63108100 1.36129700<br>C 1.06662300 2.53898700 -1.11694200<br>H 1.45171300 3.54397600 -0.89958400<br>H 0.42566500 2.61046000 -1.99894900<br>C 2.23498500 1.58932600 -1.38703000<br>H 2.96321100 2.14903200 -1.98243600<br>H 2.75620700 1.35437500 -0.44929300<br>C 1.92335100 0.31074200 -2.16891600<br>H 1.20214300 0.51796100 -2.96077500<br>H 2.84251000 -0.06485800 -2.63679900<br>C -1.94814100 0.11623000 2.39840000<br>H -1.15581500 0.62108800 2.94933900<br>H -1.77714100 -0.95675100 2.48729300<br>H -2.92258000 0.36689100 2.83386200<br>C -0.50172600 -2.90161500 0.73119100<br>H -0.58943100 -2.54104100 1.75507700<br>H 0.55315800 -3.09711700 0.53710900<br>H -1.05522500 -3.83978400 0.60893500<br>C 2.51297500 -1.42750300 -0.61215100<br>H 2.17167700 -2.17299000 0.10681700<br>H 3.08873300 -0.67492300 -0.07889200<br>H 3.16282700 -1.92111200 -1.34309500<br>Cr -0.15474100 0.03753100 -0.10547100<br>Cl -1.22127600 0.52467200 -2.16894600<br>N 1.81879800 -0.58644600 2.20399400<br>O 1.91351000 -1.48839000 3.01811800<br>O 2.70793300 0.20491400 1.89278100 | | |

| 14 TMC | | |
|---|---|---|
| ES cis | ES trans | TScis |
| Cr -0.08442400 -0.31188000 0.30637000<br>C 1.24058900 -3.08723700 -0.14351500 | Cr -0.32683100 -0.31382300 0.11829000<br>C -2.36520100 0.58061400 2.30491600 | Cr -0.19021200 0.28480600 0.25451700<br>C 1.44901000 0.17445000 2.92844500 |

| | | |
|---|---|---|
| N 1.07364000 -1.76129800 -0.78547800 | N -1.27041100 1.09859600 1.45136300 | N 1.40144500 -0.41399200 1.57035800 |
| N -1.26745800 1.28396100 1.21126600 | N 0.79568800 -1.58357600 -1.24841300 | N -1.74376300 0.83320500 -1.14171300 |
| C -1.92120400 2.02707900 0.08610200 | C 2.20819500 -1.08761700 -1.21696600 | C -2.09000600 -0.42160200 -1.87791700 |
| C -2.87772400 1.19393300 -0.76210700 | C 2.89907200 -1.19789000 0.13856600 | C -2.64375800 -1.54965500 -1.01035100 |
| C -2.33590900 0.03561600 -1.59367300 | C 2.39567400 -0.36079600 1.31033300 | C -1.77049000 -2.20422200 0.05576100 |
| N -1.76717000 -1.10429500 -0.80384700 | N 1.01191900 -0.68110800 1.78424100 | N -1.33294600 -1.30979300 1.17171100 |
| C -2.87459600 -1.77983200 -0.08212300 | C 1.02905800 -2.02247100 2.41886700 | C -2.51165800 -0.96737400 2.00469500 |
| C 2.42496600 -1.23421200 -1.14782100 | C -1.76707900 2.30504600 0.72131900 | C 2.75161100 -0.27206400 0.94477300 |
| C 3.23763600 -0.71891800 0.02767600 | C -2.69804300 1.99717600 -0.44026000 | C 3.06051900 1.12608600 0.43447400 |
| C 2.84200800 0.66012800 0.52472800 | C -2.00688900 1.53626100 -1.71128400 | C 2.41728000 1.47314700 -0.89699800 |
| N 1.57541700 0.69403300 1.31868400 | N -1.51068100 0.12625800 -1.67022900 | N 0.96643200 1.82278700 -0.80469500 |
| C 1.10202300 2.11018200 1.39348500 | C -0.52025800 -0.06701500 -2.77166900 | C 0.36378600 1.75631300 -2.16629700 |
| C -0.30342500 2.17727400 1.93696300 | C 0.20339000 -1.38212300 -2.61416100 | C -1.14189900 1.83587900 -2.08540400 |
| C 0.27030900 -1.91394200 -2.03490000 | C -0.11565900 1.49772600 2.30856800 | C 1.06407500 -1.86293300 1.66018500 |
| C -1.18645500 -2.14441500 -1.71708100 | C 0.59477500 0.28382600 2.85476600 | C -0.37782700 -2.04809800 2.06531500 |
| C 1.87271500 0.19850900 2.68367500 | C -2.66723000 -0.77596100 -1.88805200 | C 0.85517300 3.20496900 -0.28161400 |
| Cl -0.49988700 -1.73313500 2.06031900 | Cl -1.68649800 -2.05185000 0.76925800 | Cl -0.95138700 1.81044500 1.80912500 |
| O 0.48999400 0.99819000 -1.02565300 | O 0.57929400 1.25003700 -0.64291700 | O 0.74947900 -0.74673200 -0.97820700 |
| C -2.30407300 0.90993900 2.20467500 | C 0.78983800 -3.04843300 -1.01428400 | C -2.98992000 1.44832300 -0.62278900 |
| H 1.77669200 -3.75857200 -0.82638700 | H -2.69043000 1.36557700 2.99969500 | H 2.21437100 -0.33815800 3.52533800 |
| H 1.79889300 -2.98779700 0.78691900 | H -3.20610900 0.26593300 1.68799300 | H 1.68277700 1.23670300 2.86508400 |
| H 0.27336500 -3.51457800 0.11660700 | H -2.04077600 -0.29736600 2.86079300 | H 0.48134200 0.09846700 3.42119400 |
| H -2.47921300 -2.58955700 0.52848900 | H 0.02959200 -2.28446800 2.76089600 | H -2.21230200 -0.30139700 2.81251300 |
| H -3.39478400 -1.08808400 0.57480400 | H 1.34771200 -2.78515700 1.71213100 | H -3.26980800 -0.45525100 1.41606100 |
| H -3.59445700 -2.17302400 -0.81129900 | H 1.72945800 -2.01407100 3.26389100 | H -2.94807100 -1.88799300 2.41242900 |
| H 0.98187300 0.21754600 3.30897400 | H -2.35566200 -1.81888600 -1.86573800 | H -0.18754100 3.50160700 -0.18110100 |
| H 2.20517700 -0.83882700 2.64181600 | H -3.39743700 -0.65084500 -1.08849200 | H 1.29423900 3.26672800 0.71397700 |
| H 2.65761900 0.81852700 3.13543500 | H -3.13435900 -0.54542100 -2.85430800 | H 1.37848800 3.89435200 -0.95670200 |
| H -2.80056700 1.81500300 2.57759200 | H 1.37432000 -3.54899400 -1.79689700 | H -3.66645100 1.66775100 -1.45865900 |
| H -3.05333500 0.25899800 1.76194100 | H 1.22229100 -3.29317100 -0.04745700 | H -3.49645900 0.78078500 0.07033600 |
| H -1.84329000 0.36901800 3.02917000 | H -0.23562700 -3.41451400 -1.01757600 | H -2.75238800 2.36337000 -0.08288200 |
| H -2.49046400 2.85806300 0.52783400 | H 2.78349300 -1.68091100 -1.94322900 | H -2.85525200 -0.16594000 -2.62564200 |
| H -1.12670300 2.45428900 -0.52833000 | H 2.19825200 -0.05387400 -1.56999200 | H -1.19825300 -0.73754200 -2.41905200 |
| H -3.32266700 1.89065100 -1.48055700 | H 3.93405700 -0.87871500 -0.02781400 | H -2.91298600 -2.35294800 -1.70462000 |
| H -3.72568800 0.85912300 -0.15419300 | H 2.98740800 -2.24915200 0.43371800 | H -3.59899800 -1.24206800 -0.57031600 |
| H -3.16109000 -0.36656500 -2.19965700 | H 3.07689200 -0.51436500 2.15997300 | H -2.34280100 -3.03181800 0.50058400 |
| H -1.56737000 0.38236400 -2.28487100 | H 2.42029400 0.70015900 1.06439700 | H -0.87273500 -2.63555200 -0.38437000 |
| H -1.77522200 -2.19152700 -2.64219700 | H 1.47260100 0.58700300 3.43958900 | H -0.63992800 -3.11350300 2.07944800 |
| H -1.31309300 -3.10739500 -1.21565200 | H -0.06142100 -0.26403100 3.53571000 | H -0.53593100 -1.67432800 3.07979100 |
| H 0.65484200 -2.75991900 -2.62175000 | H -0.46740000 2.12384200 3.14062300 | H 1.72007900 -2.35905800 2.38943000 |
| H 0.40650800 -1.01388400 -2.63431800 | H 0.55496600 2.10653500 1.69978400 | H 1.26002200 -2.30137100 0.67866300 |
| H 2.96847400 -2.04534700 -1.65263900 | H -2.28010700 2.94379500 1.45404900 | H 3.49550700 -0.57227300 1.69597400 |
| H 2.27909400 -0.43266100 -1.87652100 | H -0.89347500 2.85098000 0.35649900 | H 2.80677400 -0.98174000 0.11470900 |
| H 4.27691000 -0.64928400 -0.30901600 | H -3.22299000 2.92643600 -0.68414200 | H 4.14414500 1.18171600 0.29077500 |
| H 3.25727300 -1.44777500 0.84655700 | H -3.48932100 1.29917100 -0.14150100 | H 2.83584200 1.88601700 1.19267900 |
| H 3.63947200 1.07349400 1.15789200 | H -2.69490100 1.61976900 -2.56401000 | H 2.93461900 2.32749600 -1.35576000 |
| H 2.71312300 1.33415500 -0.32508000 | H -1.14829700 2.17928300 -1.91379500 | H 2.50852300 0.62212200 -1.57666300 |
| H 1.77335100 2.68896900 2.04331500 | H -1.03702100 -0.04870800 -3.74166700 | H 0.73987400 2.58470200 -2.78339900 |
| H 1.15909400 2.53242500 0.38871900 | H 0.17457100 0.77320600 -2.74078600 | H 0.68777600 0.81951800 -2.62188600 |
| H -0.67084800 3.21115000 1.91074200 | H 0.99146200 -1.47470800 -3.37236100 | H -1.58537700 1.71194200 -3.08155200 |
| H -0.31792500 1.86829300 2.98531800 | H -0.48863700 -2.21154200 -2.78143500 | H -1.44568600 2.82346200 -1.73007900 |
| N 0.82299700 2.42547200 -2.89246500 | N 2.56319200 2.34637300 -1.28617700 | N 1.52957300 -2.71568800 -2.30311400 |
| O 0.40513500 1.08145500 -2.41648400 | O 1.56957800 2.11867900 -0.16580500 | O 0.46517600 -2.10997700 -1.93361800 |
| O 1.12438000 3.15928300 -2.02431000 | O 3.35802900 3.13185200 -0.93557000 | O 2.62467800 -2.38124900 -1.95709400 |
| TS1trans | INT1 | TS2 |
| Cr 0.47038500 0.02068700 0.00594900 | Cr -0.47467700 0.13732000 0.00374500 | Cr 0.19571500 0.16170900 -0.32719800 |
| C 1.52212800 2.09718900 -2.14088100 | C -2.19940500 -1.32329300 -2.18229400 | C 2.52460400 2.20496200 0.31736800 |
| N 0.27248800 1.51896000 -1.59754700 | N -0.82515300 -1.23730400 -1.64152500 | N 1.56594600 1.28642400 0.97112500 |
| N 0.53460100 -1.50562500 1.52895700 | N 0.06950600 1.53643600 1.56776400 | N -1.24053600 -0.93361100 -1.50831400 |
| C -0.62621600 -2.40774100 1.23906600 | C 1.50625400 1.88755100 1.32201400 | C -2.48904600 -0.98164600 -0.68635100 |
| C -0.58436000 -3.11195800 -0.11441400 | C 1.77187600 2.61964100 0.00975400 | C -3.10861000 0.37164000 -0.35020800 |
| C -0.63132700 -2.29721800 -1.40373200 | C 1.48082400 1.91977700 -1.31464400 | C -2.35193200 1.37056600 0.52048800 |
| N 0.53705100 -1.38528600 -1.62203600 | N 0.04100900 1.56884500 -1.54088500 | N -1.08614200 1.89597400 -0.07506500 |
| C 1.75983000 -2.19378800 -1.83705500 | C -0.74709800 2.81369600 -1.71377700 | C -1.41512600 2.75002400 -1.24130800 |
| C -0.63278300 2.64041000 -1.19824800 | C -0.35388400 -2.61152300 -1.30180100 | C 2.32293100 0.37198500 1.87537700 |
| C -0.26464000 3.30938900 0.11446800 | C -0.94223600 -3.18800100 -0.02692800 | C 3.06424100 -0.74895600 1.16510500 |
| C -0.66223500 2.53158200 1.35762400 | C -0.32711500 -2.63913600 1.24747400 | C 2.18762700 -1.91074100 0.72691600 |

| | | |
|---|---|---|
| N 0.24610400 1.39215500 1.69145700<br>C -0.44710200 0.50235400 2.66414400<br>C 0.31203400 -0.78846300 2.83026000<br>C -0.40716300 0.70528600 -2.64677000<br>C 0.33551300 -0.58584400 -2.87699200<br>C 1.48059900 1.94211100 2.29725600<br>Cl 2.76810400 0.24072200 0.01907400<br>O -1.26511300 0.18938400 -0.03654800<br>C 1.75661800 -2.32565800 1.71526400<br>H 1.28725300 2.73691400 -3.00128700<br>H 2.02398600 2.68734300 -1.37494700<br>H 2.21982800 1.31730200 -2.43943100<br>H 2.61359700 -1.53389500 -1.98150300<br>H 1.96635400 -2.82293100 -0.97540500<br>H 1.62381300 -2.83579900 -2.71630000<br>H 2.17167700 1.14542100 2.56502000<br>H 1.99822300 2.57864500 1.58002900<br>H 1.22412700 2.52843000 3.18878800<br>H 1.63063100 -2.96838500 2.59531700<br>H 1.93637500 -2.96253600 0.85244800<br>H 2.62010000 -1.67466600 1.84438400<br>H -0.64589100 -3.17886500 2.02307200<br>H -1.53383700 -1.81216300 1.34010900<br>H -1.47158600 -3.75402700 -0.13774500<br>H 0.25728500 -3.81270000 -0.14586800<br>H -0.66377700 -3.00099300 -2.24852600<br>H -1.53206400 -1.68293400 -1.44393000<br>H -0.18667200 -1.20223600 -3.61996000<br>H 1.32916200 -0.37955000 -3.28257100<br>H -0.45909200 1.27322200 -3.58628000<br>H -1.42870200 0.52123200 -2.30652400<br>H -0.62584700 3.37640700 -2.01392100<br>H -1.64438700 2.23163900 -1.12576400<br>H -0.80323100 4.26163600 0.14782800<br>H 0.79628200 3.58521000 0.13848700<br>H -0.68575600 3.20103300 2.22877500<br>H -1.66679700 2.12123500 1.22499100<br>H -0.54024400 1.00609600 3.63647500<br>H -1.45352000 0.31931500 2.27899800<br>H -0.20964100 -1.45721900 3.52660300<br>H 1.29986400 -0.59622300 3.25651400<br>N -3.76513700 0.27315600 -0.06980500<br>O -2.86723600 -0.74404600 0.06314700<br>O -4.87210700 -0.19085500 -0.02810300 | N -0.79314400 -1.27362500 1.62628300<br>C 0.14917900 -0.72709900 2.64692200<br>C -0.06848500 0.75069400 2.83740400<br>C 0.09987100 -0.67140900 -2.66788700<br>C -0.11807500 0.81007300 -2.82438400<br>C -2.15636300 -1.37504900 2.19192700<br>Cl -2.70906500 0.84412400 0.03840400<br>O 1.05932100 -0.48125100 -0.01682900<br>C -0.71220900 2.77863000 1.78065400<br>H -2.21089300 -1.97998100 -3.06184100<br>H -2.87473200 -1.71968100 -1.42370100<br>H -2.57353700 -0.33670800 -2.44881000<br>H -1.80971400 2.57322600 -1.73800500<br>H -0.57032200 3.50496500 -0.89438700<br>H -0.44713800 3.30884900 -2.64571500<br>H -2.52451400 -0.39638000 2.49428000<br>H -2.84770900 -1.74924600 1.43666700<br>H -2.14986800 -2.05603300 3.05297000<br>H -0.38707100 3.25920500 2.71188100<br>H -0.55912300 3.48175700 0.96684700<br>H -1.77367900 2.53801300 1.83049800<br>H 1.83899200 2.53005000 2.15084000<br>H 2.07789400 0.95635400 1.36160600<br>H 2.84430300 2.84185500 0.00186400<br>H 1.29327300 3.60511200 0.02631300<br>H 1.79314300 2.58551600 -2.13303600<br>H 2.05717000 0.99424600 -1.39325100<br>H 0.56897700 1.22155600 -3.57560600<br>H -1.13173400 1.01083300 -3.18216600<br>H -0.05832500 -1.17600200 -3.63124700<br>H 1.11866200 -0.88028300 -2.33410100<br>H 0.58331100 -3.26413000 -2.15589500<br>H 0.73548100 -2.56007800 -1.20631600<br>H -0.74508900 -4.26460400 -0.04075800<br>H -2.03521300 -3.10243000 -0.01407600<br>H -0.53832500 -3.31019200 2.09189600<br>H 0.75982800 -2.58383000 1.13088200<br>H 0.00765200 -1.25052100 3.60295300<br>H 1.16323300 -0.92762600 2.29336100<br>H 0.62947900 1.14765800 3.58643600<br>H -1.07720900 0.94159800 3.21448400<br>N 3.62829200 -1.58700600 -0.04175400<br>O 3.74088000 -1.10365300 -1.13673200<br>O 3.73897600 -1.15354600 1.07438600 | N 1.38781500 -1.65616800 -0.50768500<br>C 0.31297200 -2.68364900 -0.59648800<br>C -0.68539000 -2.32140700 -1.66928000<br>C 0.59414500 2.06424900 1.79080700<br>C -0.38455100 2.79312100 0.90649200<br>C 2.28432900 -1.75818600 -1.68080100<br>Cl 1.16233300 1.08813000 -2.22947900<br>O -0.24390000 -0.53132800 1.21387400<br>C -1.56695900 -0.44487000 -2.86870900<br>H 3.06813800 2.77555300 1.08163900<br>H 3.23035200 1.63619900 -0.28782300<br>H 2.01120900 2.88416000 -0.36126400<br>H -0.49629400 3.08879400 -1.71781100<br>H -1.98929600 2.19845300 -1.98108300<br>H -2.01199000 3.60913600 -0.90993900<br>H 1.74041300 -1.56715600 -2.60460600<br>H 3.06332900 -0.99834200 -1.62003900<br>H 2.74135700 -2.75581700 -1.71214800<br>H -2.32924700 -1.09365800 -3.31861800<br>H -1.94426400 0.57437800 -2.83761300<br>H -0.66704600 -0.44078600 -3.48143500<br>H -3.23337700 -1.56540400 -1.24800200<br>H -2.24906700 -1.54834500 0.21312400<br>H -4.03756900 0.14810100 0.18632400<br>H -3.44448700 0.85908700 -1.27192600<br>H -3.01580800 2.22805000 0.70611000<br>H -2.09511100 0.94271500 1.49018000<br>H -1.13148500 3.31865600 1.51516100<br>H 0.13195600 3.55592100 0.31933900<br>H 1.13119900 2.78933400 2.41863800<br>H 0.07885900 1.35744200 2.44402000<br>H 3.02912800 0.98342700 2.45489600<br>H 1.60118600 -0.05946200 2.57484400<br>H 3.79535700 -1.14941800 1.87446500<br>H 3.66280000 -0.36379500 0.33105600<br>H 2.80418000 -2.80217100 0.54501300<br>H 1.47945700 -2.15137000 1.52439600<br>H 0.74948600 -3.66830900 -0.81743000<br>H -0.16768700 -2.74096300 0.38264500<br>H -1.51045400 -3.04483000 -1.68130000<br>H -0.21797700 -2.36423700 -2.65581600<br>N -1.56085500 -1.30487700 2.47345900<br>O -1.79627600 -0.53493300 3.35837300<br>O -1.63895800 -2.49515200 2.36123100 |
| EP | | |
| Cr -0.06868100 0.00131400 -0.45042300<br>C -2.08626300 -2.18277900 -1.36526800<br>N -1.47028500 -1.62731000 -0.13586100<br>N 1.41821800 1.57510000 -0.50318500<br>C 2.35804200 1.32331500 0.63727100<br>C 3.11250100 -0.00205900 0.57610800<br>C 2.35747900 -1.32738000 0.63053400<br>N 1.41736100 -1.57291000 -0.51103900<br>C 2.20938400 -1.77521100 -1.74980300<br>C -2.56975500 -1.28095600 0.81762900<br>C -3.31368100 -0.00050900 0.48337700<br>C -2.56915500 1.27783800 0.82427000<br>N -1.46969300 1.62867500 -0.12760900<br>C -0.59883900 2.65908400 0.51754800<br>C 0.66726500 2.85499800 -0.27613800<br>C -0.60006600 -2.66150000 0.50403400<br>C 0.66612900 -2.85381500 -0.29040500<br>C -2.08568000 2.19093200 -1.35393800<br>Cl -0.29534600 0.00737000 -2.70254000<br>O -0.10065400 -0.00430900 1.54102400<br>C 2.21037500 1.78375400 -1.74080100 | | |

| | | |
|---|---|---|
| H -2.70782900 -3.04866400 -1.10506600 | | |
| H -2.70091800 -1.42510200 -1.85151500 | | |
| H -1.32447900 -2.47915600 -2.08415100 | | |
| H 1.54063000 -1.91960100 -2.59692600 | | |
| H 2.83869100 -0.91390000 -1.95957800 | | |
| H 2.85576900 -2.65329000 -1.62769700 | | |
| H -1.32390600 2.49036200 -2.07154600 | | |
| H -2.70112600 1.43626300 -1.84385000 | | |
| H -2.70642000 3.05598500 -1.08902400 | | |
| H 2.85641000 2.66146100 -1.61426200 | | |
| H 2.84011300 0.92377300 -1.95466900 | | |
| H 1.54170300 1.93206200 -2.58731800 | | |
| H 3.10114700 2.13383900 0.63062700 | | |
| H 1.79061800 1.42964600 1.56324600 | | |
| H 3.78153600 -0.00445400 1.44316700 | | |
| H 3.78939100 0.00000600 -0.28538100 | | |
| H 3.10024500 -2.13815800 0.61954200 | | |
| H 1.79014900 -1.43816800 1.55599400 | | |
| H 1.32198200 -3.57941700 0.20719600 | | |
| H 0.43980000 -3.26003400 -1.27984500 | | |
| H -1.14470000 -3.61370700 0.56714500 | | |
| H -0.37724800 -2.33264800 1.52209100 | | |
| H -3.26843400 -2.12844900 0.83098900 | | |
| H -2.12974100 -1.20600100 1.81504200 | | |
| H -4.23501500 -0.00184500 1.07394200 | | |
| H -3.64840200 0.00229500 -0.56062100 | | |
| H -3.26744000 2.12559200 0.84220500 | | |
| H -2.12894200 1.19750500 1.82121200 | | |
| H -1.14307600 3.61119000 0.58578800 | | |
| H -0.37603400 2.32474200 1.53388200 | | |
| H 1.32331800 3.57808100 0.22488100 | | |
| H 0.44083300 3.26605300 -1.26356400 | | |
| N 0.30413600 -0.00806300 2.80122900 | | |
| O 0.45902600 -1.10386700 3.33548400 | | |
| O 0.46113100 1.08494900 3.34101200 | | |

| **15 TMC** | | |
|---|---|---|
| ES cis | ES trans | TScis |
| N -1.82953500 -0.80030300 -1.08994000 | N -1.77405400 -1.55436900 -0.15697600 | N 0.87029700 2.19291400 0.10979500 |
| N 0.84303900 -1.92989500 -0.58118500 | N 1.12085500 -1.73386300 -0.71239100 | N -1.80331300 1.15005700 0.84461200 |
| C -2.16073600 -0.46379400 -2.49815100 | C -2.68079300 -1.66032600 -1.32863100 | C 0.94344500 3.10244300 -1.06028900 |
| C -3.31548600 0.99737100 -0.15210900 | C -3.34165000 -0.07812200 1.13755700 | C 3.19112900 1.29461100 -0.23228000 |
| C -3.02433300 -0.48562900 -0.25277900 | C -2.62211500 -1.40858900 1.06160100 | C 2.24451400 2.05754300 0.67036800 |
| C -0.16314300 -2.56771200 -1.47751200 | C 0.15019200 -2.81723300 -1.03448900 | C -1.45971900 2.59875100 0.78337900 |
| C -1.56370900 -2.26401100 -1.00661100 | C -0.99588000 -2.82125300 -0.05267000 | C -0.00842000 2.81993000 1.13341100 |
| C 2.16890000 -1.96417500 -1.26518700 | C 2.02854700 -1.55339500 -1.88173400 | C -3.12531200 0.95320600 0.18352400 |
| C 0.93953600 -2.72813100 0.66454800 | C 1.93533500 -2.15490300 0.45192300 | C -1.93034600 0.75542800 2.26798300 |
| N 2.05608300 0.81726500 0.39119200 | N 1.56616100 1.38424700 -0.58935500 | N -1.31913000 -1.62983600 -0.55946400 |
| N -0.96777000 1.99144500 0.17066000 | N -1.53469200 1.57976300 0.36549400 | N 1.72455300 -0.64583800 -1.06497100 |
| C 2.43178600 0.60387600 1.80766600 | C 2.49807700 1.62069400 0.53816900 | C -1.34341800 -2.60943600 0.54906400 |
| C 3.27011600 -1.26232100 -0.49878500 | C 3.01001200 -0.41109600 -1.72499100 | C -3.54126800 -0.49834100 0.06698600 |
| C 3.15867700 0.24743400 -0.45706200 | C 2.39410700 0.97137600 -1.77515600 | C -2.74431000 -1.31890900 -0.92554600 |
| C -0.20591200 2.58026800 1.31145900 | C -0.67007400 2.60339500 1.02494200 | C 1.65671600 -2.11312200 -0.81062400 |
| C 2.07409800 2.28574500 0.11159700 | C 0.96833100 2.69781800 -0.98273900 | C -0.74850100 -2.29281300 -1.77433400 |
| C -2.35041400 1.77573500 0.71614300 | C -2.45658400 1.10940800 1.45416800 | C 2.93733500 -0.19582000 -0.30242600 |
| C -1.05272400 2.96717200 -0.93766000 | C -2.35184700 2.21342100 -0.69286800 | C 1.94926100 -0.39426100 -2.50387400 |
| C 1.17051400 3.13169200 0.99063200 | C 0.21356600 3.43181400 0.11062200 | C 0.63636800 -2.89488600 -1.61607900 |
| Cl 0.63824900 0.69291900 -2.43471300 | Cl -0.65304200 0.23992400 -2.53945100 | Cl -0.90877800 1.00154200 -2.23004700 |
| Cr 0.04823400 0.07175100 -0.27664200 | Cr -0.16082900 -0.02796600 -0.27779900 | Cr -0.13227900 0.20202600 -0.17738900 |
| O -0.74254700 -0.51647700 1.37547400 | O -0.04319900 -0.46600000 1.59256700 | O 0.69121800 -0.15254000 1.42909900 |
| O -0.03378100 -0.63011400 2.58160400 | O 1.00680500 0.04318100 2.38236400 | O 0.20565000 -1.25220400 2.62956300 |
| H 1.70449900 1.05530200 2.47786600 | H 1.97133500 1.99916400 1.41078200 | H -0.33393500 -2.91969800 0.81156500 |
| H 2.47154300 -0.45975900 2.03872300 | H 2.98951800 0.69407500 0.83366300 | H -1.79783700 -2.17117800 1.43708400 |

| | | |
|---|---|---|
| H 3.41859000 1.04802900 1.99432400 | H 3.26199100 2.34794100 0.23252500 | H -1.92084900 -3.49463900 0.25129200 |
| H -0.04780900 -2.87755900 1.09569800 | H 1.29486800 -2.45084300 1.28040000 | H -1.02015100 0.98956700 2.81431100 |
| H 1.39429500 -3.70135300 0.44177900 | H 2.58376200 -2.99236200 0.16501100 | H -2.78181700 1.28103700 2.71899500 |
| H 1.54482000 -2.21181500 1.40841000 | H 2.55350300 -1.32904800 0.80356900 | H -2.08944200 -0.31799500 2.35878200 |
| H -2.17275300 0.61534600 -2.64461100 | H -3.13448900 -0.69661300 -1.55436000 | H 1.43500900 2.61510000 -1.90052800 |
| H -1.40957200 -0.86309500 -3.17533100 | H -2.12944100 -1.96249300 -2.21559000 | H -0.05262600 3.38239500 -1.39392600 |
| H -3.14373700 -0.88408200 -2.74581200 | H -3.46403800 -2.39755600 -1.11034900 | H 1.50574500 4.00256500 -0.77926800 |
| H -1.52057400 3.89610600 -0.58436200 | H -2.95534700 3.02449600 -0.26360800 | H 2.87612900 -0.88593800 -2.82936200 |
| H -0.06486300 3.18489200 -1.33874300 | H -1.71959000 2.60563800 -1.48724100 | H 1.11252100 -0.76160400 -3.09529300 |
| H -1.64532800 2.55457400 -1.75482800 | H -3.01133500 1.47404400 -1.14880500 | H 2.02403200 0.67741700 -2.68960600 |
| H -2.76387000 2.77453100 0.91705100 | H -3.08632500 1.97022500 1.72124200 | H 3.80028300 -0.68798300 -0.77435900 |
| H -2.23529600 1.26679100 1.67626200 | H -1.83028700 0.87512900 2.31824300 | H 2.83726100 -0.59920000 0.70972800 |
| H -3.42975200 1.45348900 -1.14154600 | H -3.95511600 0.09965900 0.24738200 | H 3.25021100 1.74621500 -1.22913600 |
| H -4.29964300 1.09936600 0.31652700 | H -4.06122500 -0.15084300 1.95967800 | H 4.19580800 1.41350300 0.18642900 |
| H -2.85188700 -0.90440300 0.74175200 | H -1.97499400 -1.53240500 1.93265700 | H 2.15922400 1.54464100 1.63196900 |
| H -3.88434800 -1.00977400 -0.69378600 | H -3.34893100 -2.23335600 1.06189400 | H 2.63154100 3.06982200 0.85840500 |
| H 2.43398000 -3.02009700 -1.42207900 | H 2.57163400 -2.49822000 -2.03018100 | H -3.87441200 1.51368500 0.76162500 |
| H 2.04020600 -1.49687200 -2.24552900 | H 1.39758300 -1.38378900 -2.75784900 | H -3.05889200 1.40148700 -0.81112300 |
| H 4.20820300 -1.48547800 -1.01758100 | H 3.69994000 -0.46906100 -2.57320800 | H -4.57437500 -0.50295900 -0.29572600 |
| H 3.40001300 -1.68846000 0.50255900 | H 3.64565800 -0.54286900 -0.84218500 | H -3.59235200 -0.98387300 1.04789300 |
| H 3.01576700 0.63291000 -1.46847900 | H 1.75510700 1.05596700 -2.65643700 | H -2.72061400 -0.80940400 -1.89127500 |
| H 4.09761000 0.67074400 -0.07190900 | H 3.19281800 1.72075800 -1.87198100 | H -3.24278600 -2.28733700 -1.07673900 |
| H 1.81583900 2.39090200 -0.94550500 | H 0.32411400 2.48983700 -1.84046500 | H -0.76762900 -1.53749300 -2.56401500 |
| H 3.10976300 2.63858200 0.22397000 | H 1.78173900 3.34299600 -1.34609800 | H -1.44520200 -3.08736600 -2.07955300 |
| H 1.65237000 3.33325300 1.95358800 | H 0.90938400 3.97084400 0.76274400 | H 0.57227200 -3.88554800 -1.15247300 |
| H 1.08346000 4.10948400 0.50501300 | H -0.37532200 4.21237600 -0.38282100 | H 1.01304100 -3.07906100 -2.62798600 |
| H -0.81107300 3.38988100 1.74549400 | H -1.31907100 3.28641400 1.59268700 | H 2.65190100 -2.54229900 -0.99970600 |
| H -0.15556300 1.79197100 2.06991900 | H -0.07454700 2.05748300 1.76469900 | H 1.48216200 -2.21093200 0.26520400 |
| H -1.70514000 -2.56220600 0.03640300 | H -0.63352500 -2.89886300 0.97640700 | H 0.23580400 2.36543700 2.09760500 |
| H -2.30149700 -2.81158400 -1.60849200 | H -1.65588900 -3.68002600 -0.23530500 | H 0.21173100 3.89355700 1.20901000 |
| H 0.00420300 -2.17109900 -2.48445700 | H -0.20853200 -2.63404400 -2.05269300 | H -1.66728700 2.93400700 -0.23802900 |
| H 0.01049800 -3.65195500 -1.51452800 | H 0.66550200 -3.78780700 -1.03408500 | H -2.11608300 3.16172700 1.46126500 |
| N -0.67424500 -1.59721000 3.47967900 | N 1.38784000 -1.01430500 3.36713800 | N 1.14896100 -1.94803500 3.12119300 |
| O -1.62702900 -2.11571400 3.01939600 | O 2.21927500 -0.58752600 4.07831200 | O 2.27702000 -1.91066500 2.72150500 |
| TS1trans | INT1 | TS2 |
| N 2.14596200 0.58483000 0.94613500 | N -0.24619500 2.22907700 -0.71694100 | N 1.49084800 1.81800800 0.41379100 |
| N -0.35128600 2.13841400 0.63590500 | N 1.89447000 0.29865000 -1.28082600 | N -1.45919700 1.60131400 0.66534200 |
| C 3.32821500 1.01202100 0.16045100 | C 0.08059000 3.43113500 0.08845000 | C 2.03821700 2.71360600 -0.63380200 |
| C 2.82384000 -1.83568800 0.98887500 | C -2.64759300 2.19697900 0.03637600 | C 3.44238700 0.23215800 0.33351800 |
| C 2.53884800 -0.58126000 1.78593800 | C -1.67974900 2.30332000 -1.12059200 | C 2.63712600 1.21819800 1.15408200 |
| C 0.93672100 2.72735000 1.09318800 | C 1.96395600 1.73048700 -1.67771200 | C -0.69955200 2.87775000 0.79252900 |
| C 1.75075100 1.70467300 1.84585100 | C 0.58211600 2.26035400 -1.95600700 | C 0.67811600 2.63500000 1.35757400 |
| C -0.95132000 3.05238300 -0.37507600 | C 3.21031200 -0.08703300 -0.69872700 | C -2.64470700 1.86158700 -0.19698500 |
| C -1.27164800 2.04872800 1.79577000 | C 1.64510700 -0.52130300 -2.49063700 | C -1.92398400 1.19089500 2.00960000 |
| N -1.60747200 0.03442400 -1.35369000 | N 0.94838400 -1.63933600 1.00009900 | N -1.57977600 -1.09787200 -0.90406500 |
| N 0.92090500 -1.84266600 -0.74440300 | N -1.61880600 0.27605600 1.39178800 | N 1.63315700 -1.14534100 -0.86510600 |
| C -2.76815600 -0.48008400 -0.58918000 | C 0.49555500 -2.82379000 0.23279200 | C -2.08081900 -2.03789300 0.12027400 |
| C -2.21161100 2.50979600 -1.01385000 | C 3.24604600 -1.49310500 -0.13967600 | C -3.44726100 0.62139300 -0.52200000 |
| C -1.99814700 1.35192600 -1.96439600 | C 2.44382900 -1.70180900 1.12758600 | C -2.75516400 -0.34422000 -1.45938800 |
| C -0.30396700 -2.66569700 -0.97740000 | C -1.97878000 -1.14803200 1.64630500 | C 1.09165600 -2.52647500 -0.74819900 |
| C -1.34633200 -0.86217500 -2.52414500 | C 0.43906400 -1.73043000 2.40402000 | C -1.04930800 -1.87792000 -2.06509200 |
| C 1.59737900 -2.51381200 0.41667700 | C -2.77926000 0.80219600 0.60371100 | C 2.77421800 -1.10849800 0.10887700 |
| C 1.80689700 -1.92567000 -1.92531400 | C -1.52706700 1.00975700 2.67084300 | C 2.16553300 -0.92707900 -2.22715000 |
| C -1.12283600 -2.32964800 -2.20855600 | C -1.05706500 -1.92980000 2.56310700 | C 0.01624000 -2.91071400 -1.74726900 |
| Cl 1.22678900 1.20240200 -2.00425900 | Cl 1.54807400 1.35037500 1.75961100 | Cl -0.10626300 1.34093100 -2.27415500 |
| Cr 0.23303100 0.15955400 -0.15410600 | Cr 0.21230400 0.24344700 0.12630300 | Cr 0.04015200 0.24519800 -0.19024100 |
| O -0.29005300 -0.47900200 1.34741600 | O -0.70749200 -0.47885900 -1.02192200 | O 0.30751000 -0.37012000 1.39495000 |
| O -1.52588800 -1.80551000 1.95761400 | O -3.20984400 -1.66092700 -2.22143400 | O 0.82880700 -2.51902100 2.89036500 |
| H -2.56838600 -1.47707900 -0.20332500 | H -0.58621800 -2.81244500 0.11425400 | H -1.27321400 -2.66860000 0.48850400 |
| H -2.97843800 0.16576900 0.26447900 | H 0.92847100 -2.81622100 -0.76679700 | H -2.49540000 -1.49708300 0.96983800 |
| H -3.65279300 -0.51407400 -1.23801300 | H 0.80161800 -3.74317700 0.74863300 | H -2.86132700 -2.68026500 -0.30891900 |
| H -0.80702000 1.51319000 2.61964900 | H 0.72495600 -0.21331800 -2.98168200 | H -1.08410000 1.12351500 2.69686000 |
| H -1.55291000 3.05991900 2.11616000 | H 2.49408000 -0.42617700 -3.17996800 | H -2.65831100 1.91688800 2.38212700 |
| H -2.16666400 1.48668300 1.53716100 | H 1.51721900 -1.56903000 -2.22458200 | H -2.38003300 0.20278800 1.97764900 |
| H 3.55158300 0.29681100 -0.63012900 | H -0.35449900 3.36335500 1.08391600 | H 2.47704000 2.13716700 -1.44753100 |
| H 3.14342000 1.96901600 -0.32163200 | H 1.15673200 3.51715200 0.22007200 | H 1.24930600 3.32310900 -1.06929100 |
| H 4.19193400 1.09893500 0.83221400 | H -0.30410900 4.32161000 -0.42507800 | H 2.80091700 3.36389800 -0.18619800 |

| | | |
|---|---|---|
| H 2.06180600 -2.97523600 -2.12414600<br>H 1.32267700 -1.49329600 -2.79896700<br>H 2.72111900 -1.35717500 -1.75283100<br>H 1.86975800 -3.52253600 0.07460800<br>H 0.84086100 -2.62063500 1.19886500<br>H 3.59028000 -1.66536700 0.22477800<br>H 3.27541900 -2.55613100 1.67837800<br>H 1.71984300 -0.76862600 2.48542700<br>H 3.42539500 -0.29148100 2.36861700<br>H -1.16482100 4.00962000 0.12283700<br>H -0.19283400 3.23351300 -1.14142000<br>H -2.63895400 3.31959900 -1.61424700<br>H -2.97982300 2.28521900 -0.26560800<br>H -1.22193900 1.60468900 -2.69001600<br>H -2.92717700 1.16576200 -2.52142600<br>H -0.48450600 -0.43014000 -3.03939900<br>H -2.20154300 -0.77526200 -3.20937500<br>H -2.08242900 -2.84408700 -2.08735100<br>H -0.67061000 -2.77395200 -3.10168700<br>H 0.00428700 -3.71949800 -1.03885700<br>H -0.89950000 -2.57400300 -0.06597000<br>H 1.17276700 1.27588600 2.66941900<br>H 2.64801800 2.16558100 2.28039700<br>H 1.47199600 3.07285400 0.20282500<br>H 0.73849200 3.60564800 1.72296800<br>N -2.43105600 -1.10033800 2.66085800<br>O -3.25888800 -1.86798100 3.08871100 | H -2.47222400 0.92248100 3.22380200<br>H -0.70765900 0.62347500 3.27475900<br>H -1.31407300 2.06310700 2.48567900<br>H -3.65827900 0.75525900 1.26263500<br>H -2.94163100 0.09740500 -0.21710200<br>H -2.43548500 2.93975200 0.81324800<br>H -3.63653400 2.46800000 -0.34708400<br>H -1.85672700 1.48344000 -1.82581500<br>H -1.82645600 3.25275600 -1.65577300<br>H 3.97106800 0.02297100 -1.48566700<br>H 3.43292200 0.63340300 0.09277900<br>H 4.28772100 -1.70337500 0.12393600<br>H 3.00011900 -2.23870500 -0.90415700<br>H 2.72576000 -0.95309500 1.87212600<br>H 2.67682600 -2.69164600 1.54510900<br>H 0.77638000 -0.81672900 2.90093200<br>H 0.95686900 -2.56806800 2.89323400<br>H -1.31653800 -2.98602600 2.43061500<br>H -1.28750600 -1.70929900 3.61100900<br>H -2.99390400 -1.17191900 2.06975400<br>H -2.04252400 -1.60876200 0.65515400<br>H 0.07411000 1.64815800 -2.70639600<br>H 0.62471700 3.28632200 -2.34453200<br>H 2.43296000 2.27558900 -0.85199900<br>H 2.60988300 1.84116100 -2.55977200<br>N -2.25969600 -2.37727500 -2.04649100<br>O -1.39767100 -2.80899800 -2.76443200 | H 2.94783800 -1.66626400 -2.44698600<br>H 1.37276100 -1.00288700 -2.96892800<br>H 2.57985300 0.07793600 -2.30974000<br>H 3.52039700 -1.82910100 -0.25612500<br>H 2.38106100 -1.47933500 1.05910200<br>H 3.79000200 0.67761500 -0.60525100<br>H 4.35795500 0.02360800 0.89664400<br>H 2.22827300 0.71756900 2.03590000<br>H 3.28228100 2.04060000 1.49622500<br>H -3.27868500 2.60084800 0.31446200<br>H -2.27668900 2.31267300 -1.12267700<br>H -4.35311800 0.95299300 -1.03989500<br>H -3.81226900 0.12283000 0.38328300<br>H -2.39483600 0.19231400 -2.34093500<br>H -3.47586100 -1.10219600 -1.79890300<br>H -0.68696100 -1.13449100 -2.78004000<br>H -1.89859900 -2.38877700 -2.54174000<br>H -0.44278200 -3.83263400 -1.37398700<br>H 0.47303900 -3.18714000 -2.70366600<br>H 1.92916500 -3.23334300 -0.84575900<br>H 0.74116400 -2.61302600 0.28480300<br>H 0.62486100 2.08795600 2.30330500<br>H 1.18645600 3.58814600 1.55692900<br>H -0.63241200 3.31257100 -0.21028400<br>H -1.26031600 3.58075600 1.42436200<br>N -0.16558100 -1.89646600 2.65866600<br>O -1.22719100 -1.78404000 3.19886700 |
| EP | | |
| N 1.24700600 1.98043600 -0.00435200<br>N -1.65006800 1.53605200 0.28923800<br>C 1.71969000 2.63480200 -1.25228700<br>C 3.38913900 0.70653300 0.29044400<br>C 2.43720500 1.73411600 0.86207300<br>C -1.04192400 2.87749500 0.07556400<br>C 0.33401400 2.92969400 0.69285600<br>C -2.84587500 1.40772000 -0.59206400<br>C -2.11516900 1.45626300 1.69670800<br>N -1.41359400 -1.50036600 -0.54972500<br>N 1.77402700 -1.09864800 -0.58448900<br>C -1.77281400 -2.21035300 0.69894300<br>C -3.48207700 0.03441100 -0.54622200<br>C -2.68513900 -1.05891100 -1.22301500<br>C 1.42234500 -2.49334800 -0.17520300<br>C -0.79693600 -2.45951700 -1.52024600<br>C 2.89188500 -0.72074300 0.35018500<br>C 2.30246900 -1.11820400 -1.96687400<br>C 0.41456500 -3.23165800 -1.03396000<br>Cl -0.25799900 0.68213400 -2.45970900<br>Cr -0.00416700 0.16090200 -0.24036400<br>O 0.28043700 -0.04484100 1.70210300<br>O 0.94313000 -1.75882600 2.90173800<br>H -0.88815200 -2.63360700 1.17559300<br>H -2.24251100 -1.52481800 1.40556000<br>H -2.47178100 -3.02763800 0.47974500<br>H -1.30834200 1.67956300 2.38822100<br>H -2.93985500 2.16546800 1.84174800<br>H -2.45256500 0.45192900 1.94084900<br>H 2.21450100 1.91829400 -1.90513600<br>H 0.88111800 3.04257700 -1.81225300<br>H 2.41526800 3.44253700 -0.99202600<br>H 3.16097100 -1.80070000 -2.02184500<br>H 1.53404700 -1.43932800 -2.66831600<br>H 2.61917900 -0.11951700 -2.26860100<br>H 3.72462200 -1.40140700 0.12255000<br>H 2.54869400 -0.95432600 1.36153100<br>H 3.72139200 0.98211900 -0.71645100<br>H 4.29744000 0.73650700 0.90076900 | | |

| | | |
|---|---|---|
| H 2.07337100 1.40435800 1.83837800<br>H 2.95332100 2.69507000 1.00062000<br>H -3.57363100 2.17091500 -0.28096400<br>H -2.53040000 1.64008000 -1.61234400<br>H -4.42822700 0.10130700 -1.09320300<br>H -3.77056100 -0.24420900 0.47308700<br>H -2.41150600 -0.74242300 -2.23245500<br>H -3.31037700 -1.95812200 -1.31541800<br>H -0.55523500 -1.86585800 -2.40564600<br>H -1.57470700 -3.17595100 -1.82038000<br>H 0.10238100 -4.10468800 -0.45053400<br>H 0.89729400 -3.64223900 -1.92743700<br>H 2.35330200 -3.07832600 -0.14949400<br>H 1.09080700 -2.42416200 0.86259400<br>H 0.30131200 2.64096300 1.74711700<br>H 0.74739100 3.94587900 0.64422000<br>H -0.99251300 3.04617400 -1.00492900<br>H -1.69222400 3.65200200 0.50473700<br>N 0.08850200 -0.89460500 2.72882900<br>O -0.89321700 -0.71211000 3.43065500 | | |
